# The Spruce Budworm Genome: Reconstructing the Evolutionary History of Antifreeze Proteins

**DOI:** 10.1093/gbe/evac087

**Published:** 2022-06-07

**Authors:** Catherine Béliveau, Patrick Gagné, Sandrine Picq, Oksana Vernygora, Christopher I Keeling, Kristine Pinkney, Daniel Doucet, Fayuan Wen, J Spencer Johnston, Halim Maaroufi, Brian Boyle, Jérôme Laroche, Ken Dewar, Nikoleta Juretic, Gwylim Blackburn, Audrey Nisole, Bryan Brunet, Marcelo Brandão, Lisa Lumley, Jun Duan, Guoxing Quan, Christopher J Lucarotti, Amanda D Roe, Felix A H Sperling, Roger C Levesque, Michel Cusson

**Affiliations:** Laurentian Forestry Centre, Natural Resources Canada, Quebec City, Quebec, Canada; Laurentian Forestry Centre, Natural Resources Canada, Quebec City, Quebec, Canada; Laurentian Forestry Centre, Natural Resources Canada, Quebec City, Quebec, Canada; Department of Entomology, University of Kentucky, Lexington, Kentucky, USA; Laurentian Forestry Centre, Natural Resources Canada, Quebec City, Quebec, Canada; Département de biochimie, de microbiologie et de bio-informatique, Université Laval, Quebec City, Quebec, Canada; Great Lakes Forestry Centre, Natural Resources Canada, Sault Ste. Marie, Ontario, Canada; Great Lakes Forestry Centre, Natural Resources Canada, Sault Ste. Marie, Ontario, Canada; Great Lakes Forestry Centre, Natural Resources Canada, Sault Ste. Marie, Ontario, Canada; Center for Sickle Cell Disease, College of Medicine, Howard University, Washington DC, USA; Department of Entomology, Texas A&M University, 2475 College Station, Texas, USA; Institut de biologie intégrative et des systèmes, Université Laval, Quebec City, Quebec, Canada; Institut de biologie intégrative et des systèmes, Université Laval, Quebec City, Quebec, Canada; Institut de biologie intégrative et des systèmes, Université Laval, Quebec City, Quebec, Canada; Quantitative Life Sciences, McGill University, Montreal, Quebec, Canada; Research Institute of the McGill University Health Centre, Montreal, Quebec, Canada; Pacific Forestry Centre, Natural Resources Canada, Victoria, British Columbia, Canada; Laurentian Forestry Centre, Natural Resources Canada, Quebec City, Quebec, Canada; Ottawa Research and Development Centre, Agriculture and Agri-Food Canada, Ottawa, Ontario, Canada; Laboratório de Biologia Integrativa e Sistêmica - CBMEG/UNICAMP, Campinas, Brazil; Alberta Biodiversity Monitoring Institute, University of Alberta, Edmonton, Alberta, Canada; Department of Biological Sciences, University of Alberta, Edmonton, Alberta, Canada; Great Lakes Forestry Centre, Natural Resources Canada, Sault Ste. Marie, Ontario, Canada; University of British Columbia, Vancouver, British Columbia, Canada; Great Lakes Forestry Centre, Natural Resources Canada, Sault Ste. Marie, Ontario, Canada; Atlantic Forestry Centre, Natural Resources Canada, Fredericton, New Brunswick, Canada; Great Lakes Forestry Centre, Natural Resources Canada, Sault Ste. Marie, Ontario, Canada; Department of Biological Sciences, University of Alberta, Edmonton, Alberta, Canada; Institut de biologie intégrative et des systèmes, Université Laval, Quebec City, Quebec, Canada; Laurentian Forestry Centre, Natural Resources Canada, Quebec City, Quebec, Canada; Département de biochimie, de microbiologie et de bio-informatique, Université Laval, Quebec City, Quebec, Canada; Institut de biologie intégrative et des systèmes, Université Laval, Quebec City, Quebec, Canada

**Keywords:** antifreeze proteins, evolutionary history, *Choristoneura fumiferana*, Tortricidae, genome assembly, comparative genomics

## Abstract

Insects have developed various adaptations to survive harsh winter conditions. Among freeze-intolerant species, some produce “antifreeze proteins” (AFPs) that bind to nascent ice crystals and inhibit further ice growth. Such is the case of the spruce budworm, *Choristoneura fumiferana* (Lepidoptera: Tortricidae), a destructive North American conifer pest that can withstand temperatures below −30°C. Despite the potential importance of AFPs in the adaptive diversification of *Choristoneura*, genomic tools to explore their origins have until now been limited. Here, we present a chromosome-scale genome assembly for *C. fumiferana*, which we used to conduct comparative genomic analyses aimed at reconstructing the evolutionary history of tortricid AFPs. The budworm genome features 16 genes homologous to previously reported *C. fumiferana* AFPs (CfAFPs), 15 of which map to a single region on chromosome 18. Fourteen of these were also detected in five congeneric species, indicating *Choristoneura* AFP diversification occurred before the speciation event that led to *C. fumiferana*. Although budworm AFPs were previously considered unique to the genus *Choristoneura*, a search for homologs targeting recently sequenced tortricid genomes identified seven CfAFP-like genes in the distantly related *Notocelia uddmanniana*. High structural similarity between *Notocelia* and *Choristoneura* AFPs suggests a common origin, despite the absence of homologs in three related tortricids. Interestingly, one *Notocelia* AFP formed the C-terminus of a “zonadhesin-like” protein, possibly representing the ancestral condition from which tortricid AFPs evolved. Future work should clarify the evolutionary path of AFPs between *Notocelia* and *Choristoneura* and assess the role of the “zonadhesin-like” protein as precursor of tortricid AFPs.

Significance

Some insects produce “antifreeze proteins” (AFPs) that inhibit ice crystal growth, thus allowing them to avoid freezing during the winter. The spruce budworm, a tortricid moth, produces different isoforms of a unique AFP type whose evolutionary origins are unknown. We used an assembly of this species' genome to map its AFPs and to conduct comparative genomic analyses aimed at reconstructing their evolutionary history. Although found in congeneric species, homologs of budworm AFPs were detected in only one of four tortricid species known to display varying degrees of relatedness to the budworm. Unexpectedly, one AFP sequence discovered in this moth formed the end portion of a longer, seemingly unrelated protein that may represent the ancestral condition from which tortricid AFPs evolved.

## Introduction

Insects have evolved various physiological mechanisms to help them survive cold winter temperatures. Those that are freeze-intolerant typically depress their freezing point through the production of cryoprotectants (e.g. glycerol), thereby inducing “supercooling” in a solute concentration-dependent manner. This phenomenon enables the animal to remain in an unfrozen state at temperatures well below 0°C. In some freeze-avoiding species, further depression of their freezing point is achieved by the production of proteins that bind to nascent ice crystals, resulting in inhibition of ice growth (Teets and Denlinger 2013). Known as AFPs or ice-binding proteins (IBPs), these molecules induce thermal hysteresis, a term that refers to the difference between freezing and melting points in the presence of AFPs (Cheng 1998). AFPs have evolved independently in diverse organisms including fishes, insects, marine invertebrates, plants, fungi, and bacteria (Duman and Serianni 2002; Box et al. 2022). Among animals, insect AFPs are up to 100 times more active than those of fishes (Scotter et al. 2006), which have lower thermal hysteresis requirements than terrestrial arthropods (freezing point of sea water: −1.86°C; De Vries 1986).

AFPs have been reported in numerous hexapod species in the orders Coleoptera, Lepidoptera, Hemiptera, Diptera (Duman and Newton 2020), and Collembola (Graham and Davies 2005). Although these proteins tend to have some structural features in common across all taxa (e.g. flat ice-binding surfaces), most show no primary sequence homology (except some AFPs within Coleoptera; Cheng 1998) and are thus considered to have distinct evolutionary origins (Box et al. 2022). Among the best characterized insect AFPs are those of the spruce budworm, *Choristoneura fumiferana* (Lepidoptera: Tortricidae), a highly destructive defoliator of fir and spruce forests in Canada and the northeastern United States (Johns et al. 2019).

In late summer, *C. fumiferana* first-instar larvae spin a thin silk hibernaculum on host tree branches (usually in flower scars), within which they molt to the second instar. These young larvae then enter diapause, a state of arrested development and suppressed metabolism that is key to surviving during the long winter months in the boreal forest. Development and molting to subsequent larval stadia only resume in the spring. As a result, the spruce budworm spends most of its 1-year life cycle as a tiny caterpillar, in the tree canopy, fully exposed to harsh winter conditions (supplementary fig S1, Supplementary Material online). Its ability to supercool during that period has been well documented, with the production of glycerol playing a major role in this process (Han and Bauce 1993). Glycerol alone is apparently not sufficient, however. AFP production is thought to be critical to maintaining a supercooled state at temperatures that plunge well below-20°C over periods of several days (Marshall and Roe 2021).

Evidence for the presence of AFPs in *C. fumiferana* was first provided by Hew et al. (1983), who purified from second-instar larvae a low-molecular weight, cysteine-rich protein displaying thermal hysteresis (TH) activity. While a budworm AFP cDNA was subsequently isolated and shown to encode a 9 kDa threonine-, serine-, and cysteine-rich protein exhibiting substantial TH activity (5–10°C; Tyshenko et al. 1997), several additional *C. fumiferana* AFPs (CfAFPs) were eventually characterized (Doucet et al. 2000, 2002), leading to the recognition of two isoform types, the “short” (∼9 kDa) and “long” (∼12 kDa) isoforms. Doucet et al. (2000, 2002) also used Southern analyses to show that the *C. fumiferana* genome likely encoded ∼17 paralogous AFPs, while Qin et al. (2007) revealed via Northern analyses temporal and tissue-specific differences in the expression of selected isoforms. Structural characterization of these proteins, using molecular modeling (Doucet et al. 2000), NMR spectroscopy (Gauthier et al. 1998; Graether et al. 2000), and X-ray crystallography (Leinala et al. 2000, 2002) revealed a left-handed β-helical structure consisting of 15-amino acid coils, each featuring a Thr-X-Thr (TXT) motif on one face of the triangular helix. The regular array of TXT motifs was found to be instrumental in binding to both prism and basal planes of ice crystals, a feature deemed responsible for the “hyperactivity” of CfAFPs relative to those of fishes, which do not bind to the basal plane (Pertaya et al. 2008). Finally, long isoforms of CfAFPs were found to contain a 31-amino acid insertion resulting in the addition of two TXT motifs (7 instead of 5 in the short isoforms) and a tripling of TH activity relative to that of short isoforms (Leinala et al. 2002).

Since AFPs have evolved independently in different groups of organisms, each displaying a unique primary sequence and protein fold, they are expected to have arisen through different mechanisms, including neofunctionalization of unrelated ancestral proteins and *de novo* gene birth (Cheng 1998). Most investigations conducted to date have focused on the evolutionary origins of different classes of fish AFPs, and these have now been elucidated in several taxa. For example, type-II fish AFPs, found in Atlantic herring, sea raven and rainbow smelt, evolved from a progenitor Ca^2+^-dependent C-type lectin, raising the possibility that mechanisms involved in binding ice may be similar to those responsible for binding carbohydrates (Ewart and Fletcher 1993; Loewen et al. 1998; Liu et al. 2007). Interestingly, the presence of homologous type-II AFPs in phylogenetically distant fish taxa has been shown to be the outcome of horizontal gene transfer (HGT), from herring to smelt (Graham et al. 2008, 2012; Graham and Davies 2021). Similarly, type-III AFPs found in Antarctic zoarcid fishes, are considered to be derived from a sialic acid synthase (SAS) gene whose ancestral product displayed both SAS and rudimentary ice-binding activity, with the latter function having been optimized in one copy of the gene following a duplication event (Baardsnen and Davies 2001; Deng et al. 2010). In Antarctic notothenioid fishes, on the other hand, *de novo* gene birth was identified as the mechanism that gave rise to their antifreeze glycoproteins (AFGPs), which are made up of varying numbers of Thr-Ala-Ala repeats, where the Thr residues are glycosylated. Indeed, the repetitive AFGP coding region appears to have been generated from duplications of a partly non-sense 9-nt sequence that straddled an intron-exon junction within a trypsinogen-like protease gene (Chen et al. 1997a). For insect AFPs, however, their evolutionary origin has yet to be addressed.

Here, we use a chromosome-scale assembly of the *C. fumiferana* genome to identify and map all CfAFPs and to conduct comparative genomic analyses of congeneric species and other tortricids, in order to reconstruct the evolutionary history of these intriguing molecules and find clues to their initial origin. The present assembly, based primarily on PacBio long reads, is distinct from a more fragmented Roche 454-based assembly we released earlier (referred to as “bw6”; doi.org/10.5061/dryad.1vr6g3f) and used as reference for several population genomics and phylogenetic analyses (Brunet et al. 2017; Dupuis et al. 2017; Blackburn et al. 2017; Larroque et al. 2019; Lumley et al. 2020; French et al. 2020; Nelson et al. 2022), as well as to develop a *C. fumiferana* linkage map (Picq et al. 2018) and to identify antimicrobial peptides unique to the budworm (Maaroufi et al. 2021).

## Results

### Overall Features of the Spruce Budworm Genome Assembly

Hi-Rise scaffolding of the Canu/SSPACE PacBio-based assembly yielded 30 chromosome-scale scaffolds, plus 304 smaller ones, the latter representing 1% of the total assembly. The chromosome-length scaffolds each corresponded to one of the 30 linkage groups identified by Picq et al. (2018; see supplementary file 1, Supplementary Material online). Final assembly size (570 Mb for the 30 chromosome-length scaffolds; table 1) was ∼9% larger than the genome size estimated using flow cytometry (∼520 Mb; 516.4 ± 5.5 Mb for males and 523.3 ± 1.6 Mb for females). The presence of duplicated allelic variants remaining after the Purge Haplotig step (see duplicated BUSCOs in table 1, and Materials and Methods section for details on assembly and scaffolding steps) appears to account, at least in part, for the observed difference in genome size estimates.

**Table 1:** Summary of Genome Assembly Metrics and BUSCO Scores Computed at Each Stage of the Assembly, Scaffolding, and Polishing Process

	Original Canu Assembly	First Pilon Polishing	Purge Haplotigs Step	SSPACE Scaffolding	Hi-Rise Scaffolding^a^	Second Pilon Polishing^a^
N50	50,074 bp	49,914 bp	111,073 bp	253,632 bp	20,637,981 bp	20,615,616 bp
No. of contigs	26,732	26,732	7,829	3,685	30	30
Assembly size	964.7 Mb	963.7 Mb	564.5 Mb	577.5 Mb	570.7 Mb	569.6 Mb
Complete + fragmented BUSCOs^b^	97.3 + 1.0 = 98.3%	98.2 + 0.7 = 98.9%	96.2 + 1.3 = 97.4%	96.4 + 1.1 = 97.5%	96.4 + 1.0 = 97.4%	97.2 + 0.5 = 97.7%
Duplicated BUSCOs^b^	31.8%	34.2%	7.4%	7.4%	3.3%	3.9%

a Metrics for the 30 chromosome-scale scaffolds only.

b BUSCO v.5.1.3 (Simão et al. 2015) was run using the lepidoptera_odb10.2019-11-20 reference gene set (5286 genes).

With the notable exception of *Lymantria dispar* (Hébert et al. 2019; Zhang et al. 2019; Sparks et al. 2021), the spruce budworm genome displayed a higher proportion of repetitive elements (51.75%; see supplementary table S1, Supplementary Material online) than most other published lepidopteran genomes (see, for example, Cong et al. 2016). While 13.71% of the *C. fumiferana* repetitive elements belonged to the retroelement category, the majority (36.54%) were considered unclassified (see supplementary table S1, Supplementary Material online). Although larger than many other lepidopteran genomes, the present assembly is smaller than those of three tortricid genomes that are publicly available [*N. uddmanniana* (791 Mb), *A. turbidana* (698 Mb), *C. pomonella* (773 Mb)]. The 30 chromosome-length scaffolds varied in size from ∼9.4 Mb for Chr. 28 to ∼45.8 Mb for the Z chromosome (= Chr. 1), the latter being almost twice as large as the largest autosome, Chr. 18 (∼24 Mb; see supplementary file 1, Supplementary Material online). Assessment of assembly quality using BUSCO pointed to a high degree of completeness (97.7%), based on a query dataset of 5286 lepidopteran single-copy orthologs, and an N50 of 20.6 Mb (table 1). MAKER annotation of the *C. fumiferana* genome led to the identification of 16,037 unique protein-coding genes (PCGs). In an OrthoMCL comparison of *C. fumiferana*’s proteome with those of *B. mori*, *P. machaon*, and *P. xylostella*, the spruce budworm featured the highest number of unique orthologous groups (see supplementary fig. S2, Supplementary Material online), among which AFPs formed a prominent category.

### Most CfAFPs Are Found within a Single Chromosomal Region

**Fig. 1.**
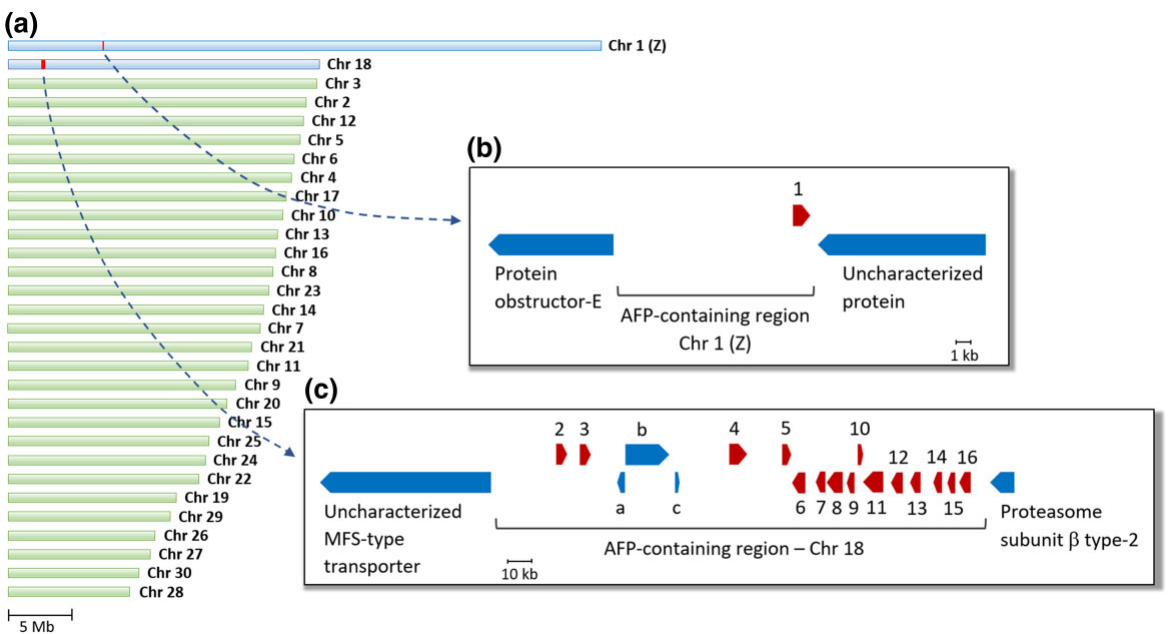
AFP-containing regions in the genome of *C. fumiferana*. (*a*) Graphic representation of *C. fumiferana*’s 30 chromosomes, in descending order of size; genes encoding CfAFPs were found on the two largest chromosomes, Chr. 1 (Z) and Chr. 18. (*b*) The AFP-containing region on Chr. 1 (Z) contains a single AFP gene, CfAFP-1 (red arrow). (*c*) The AFP-containing region on Chr. 18 contains the remaining 15 CfAFP genes (red arrows), one of which appears to be a pseudogene (CfAFP-10). Three small non-AFP genes (uncharacterized proteins; blue arrows; a, b, c) are located between CfAFP-3 and CfAFP-4. Loci are drawn to scale, with each arrow diagram representing both exons and introns of a gene. Arrows pointing in different directions represent genes found on opposite DNA strands.

Annotation of the spruce budworm genome led to the identification of 16 AFPs, including nine previously unreported isoforms. Fifteen of these (CfAFP-2 to CfAFP-16) were found within a single AFP-containing region on the second largest chromosome (Chr. 18), intermingled with three non-AFP genes; CfAFP-1, however, was found alone on the Z chromosome (fig. 1). The smallest member of this gene family, CfAFP-10, appears to be a pseudogene, as it encodes a partial protein and it is not among the 12 AFP genes for which we found transcriptomic evidence (fig. 1; see supplementary file 2, Supplementary Material online).

### Orthologs of CfAFPs Are Found in *Choristoneura* Species Related to *C. fumiferana*

**Fig. 2.**
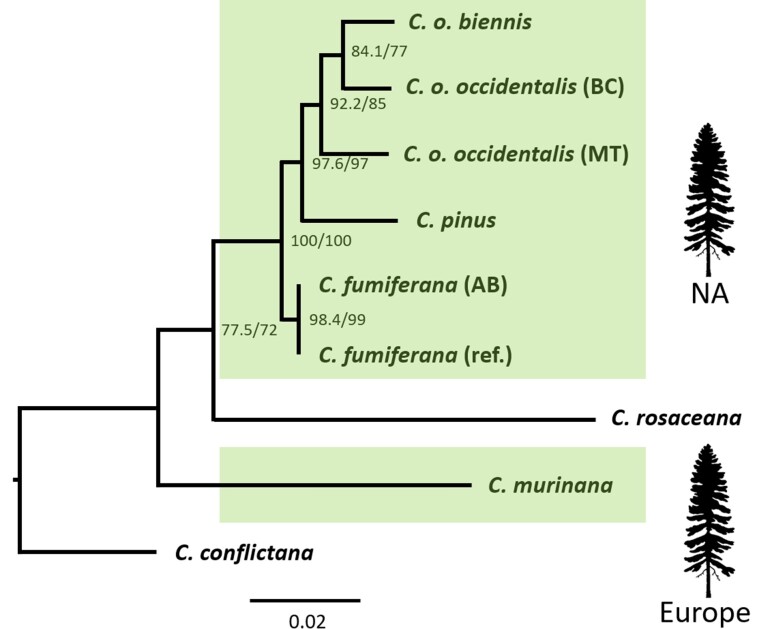
ML phylogeny of seven *Choristoneura* species/subspecies based on concatenated AFP sequences. ML tree constructed using the IQ-TREE software and concatenated nucleotide sequences of AFPs from 14 orthologous groups. The analysis includes an eastern (Ontario; the present assembly; “ref.”) and a western (Alberta; “AB”) specimen of *C. fumiferana*, and three specimens of *C. occidentalis*, including one from a southern location (Bitterroot, Montana; subspecies *occidentalis*; “MT”), one from a more northern location (Merritt, BC; subspecies *occidentalis*; “BC”), and a third one from a location further north (St. James Valley, BC; subspecies *biennis*). All species except the Nearctic *C. rosaceana* and *C. conflictana* are conifer feeders (green shading), either in North America (NA) or in Europe. ML analyses conducted on the amino acid sequences, as well as Bayesian analyses using either amino acids or nucleotides, generated trees with almost identical topologies (see supplementary file 9, Supplementary Material online). Numbers at the nodes indicate SH-aLRT/ultrafast bootstrap support values. Note: instability has been reported (Fagua et al. 2018, 2019; Dupuis et al. 2017) for the internal phylogeny of the *C. fumiferana* species complex (which here includes *C. fumiferana*, *C. occidentalis occidentalis*, *C. o. biennis*, and *C. pinus*), particularly with respect to the question of whether *C. fumiferana* or *C. pinus* forms the first divergence from the rest of the group.

To begin exploring the evolutionary history of spruce budworm AFPs, we searched for the presence of their orthologs in other *Choristoneura* species and subspecies, including closely related North American conifer feeders (*C. pinus, C. occidentalis occidentalis, C. occidentalis biennis*) and more distantly related broad-leaf pests (*C. rosaceana*, *C. conflictana*), plus a conifer feeder native to Europe (*C. murinana*). The Illumina reads generated for the above species were mapped onto our *C. fumiferana* genome assembly, which led to the reconstruction of orthologs for 14 of the 16 CfAFPs (CfAFP-10 and -11 excepted), although recovery of full sequences was not always achieved for the more distantly related taxa (primarily *C. murinana* and *C. conflictana*; see supplementary file 3, Supplementary Material online, for amino acid and nucleotide sequences). A maximum likelihood (ML) phylogenetic tree generated for the seven taxa considered here, based on the concatenated sequences of 14 AFP paralogs, produced a topology that is generally congruent with previously characterized phylogenetic relationships among these species (fig. 2; Fagua et al. 2018, 2019; Dupuis et al. 2017) and indicated that AFPs formed a large gene family in congeneric ancestors of *C. fumiferana*. The taxa represented in the tree included two geographically distinct sources (Alberta [western] and Ontario [eastern; “ref”]) for *C. fumiferana* and three for *C. occidentalis* (three different latitudes). While the two *C. fumiferana* sources featured identical nucleotide sequences for all homologous AFP pairs, some AFP homologs in the three *C. occidentalis* exemplars displayed as many as 10 amino acid substitutions, an observation that was reflected in the tree’s branching pattern (fig. 2).

**Fig. 3.**
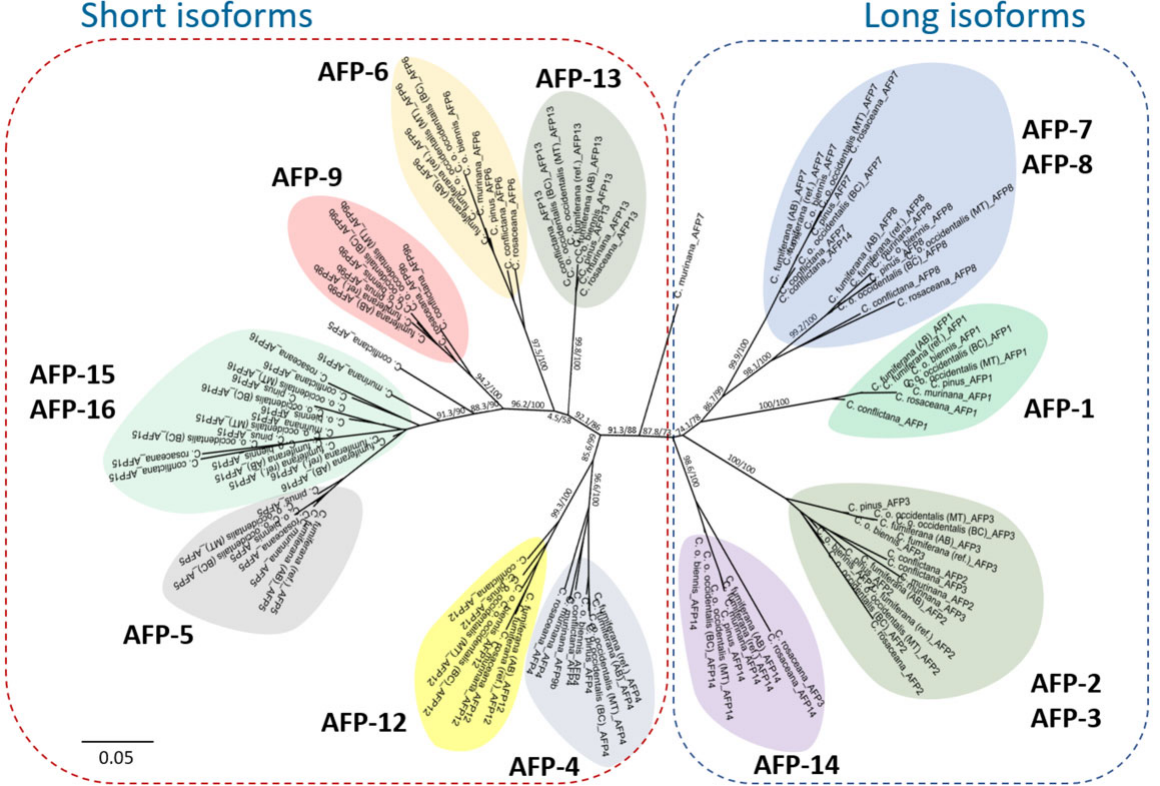
ML phylogeny of AFP orthologs among different *Choristoneura* species. ML tree constructed using the IQ-TREE software and nucleotide sequences of orthologs of 14 CfAFPs identified in seven *Choristoneura* species and subspecies. The AFP clustering pattern confirmed presumed orthologous relationships for most of them. See caption of fig. 2 for a description of the taxa and specimens used in this analysis. ML analyses conducted on the amino acid sequences, as well as Bayesian analyses using either amino acids or nucleotides, generated trees with almost identical topologies (see supplementary file 9, Supplementary Material online). Numbers at the nodes indicate SH-aLRT/ultrafast bootstrap support values.

In a phylogenetic analysis of individual AFP isoforms across these seven *Choristoneura* species and subspecies, the AFP clustering pattern confirmed presumed orthologous relationships for most of them, with exceptions seen only in the more distantly related *C. murinana* (AFP-7), *C. rosaceana* (AFP-3), and *C. conflictana* (AFP-5; fig. 3). While genes encoding short and long AFP isoforms formed two distinct groupings, some gene pairs, in both groups, displayed a closer relationship than others (e.g. AFP-2 and -3; AFP-7 and -8; AFP-15 and -16), in line with their physical proximity within the AFP-containing region (fig. 1), suggesting they are the products of recent gene duplication events. One of the newly discovered AFP genes, CfAFP-9, was found to encode a larger protein than the remaining AFPs (see supplementary file 2, Supplementary Material online), but grouped with the short isoforms; indeed, this protein lacks the 30 amino-acid insert seen in the long isoforms (Doucet et al. 2002), but instead displays an N-terminal extension, perhaps representing a new isoform type.

### *Choristoneura* AFP Homologs Are Not Unique to This Taxon

**Fig. 4.**
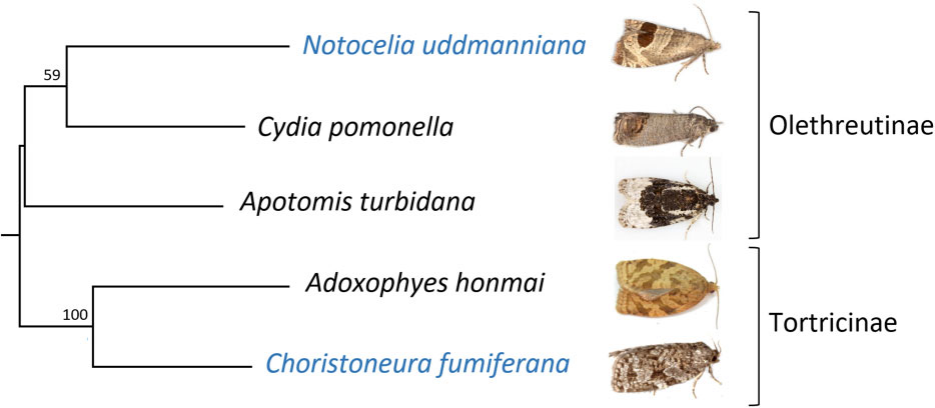
Tortricid phylogenetic tree. ML tree constructed for the five tortricid species whose genomes were searched for AFPs. The tree is based on the concatenated nucleotide sequences of the 13 mitochondrial PCGs. Numbers at the nodes indicate bootstrap support values for 2000 replicates. See Materials and Methods section for details. Moth images are from https://britishlepidoptera.weebly.com (Nu, Cp, At), http://insecta.pro/gallery (Ah), and https://bugguide.net (Cf).

Spruce budworm AFPs have so far been considered to be unique to *C. fumiferana* and a few closely related congeners (Tyshenko et al. 2005). Indeed, blast searches using CfAFPs as queries against the NCBI non-redundant database have until now supported this view. However, as one of our goals was to probe the evolutionary origins of budworm AFPs, we asked whether recently released tortricid genome assemblies, either unannotated or only partially so, could harbor AFP coding sequences not yet cataloged in public databases. To this end, we conducted local blast searches against four such assemblies (for assembly completeness statistics, see supplementary file 4, Supplementary Material online), which led to the identification of clear CfAFP homologs in the genome of *Notocelia uddmanniana*, a species distantly related to *C. fumiferana* (it belongs to a different subfamily; fig. 4); no AFPs could be detected in any of the other three species.

**Fig. 5.**
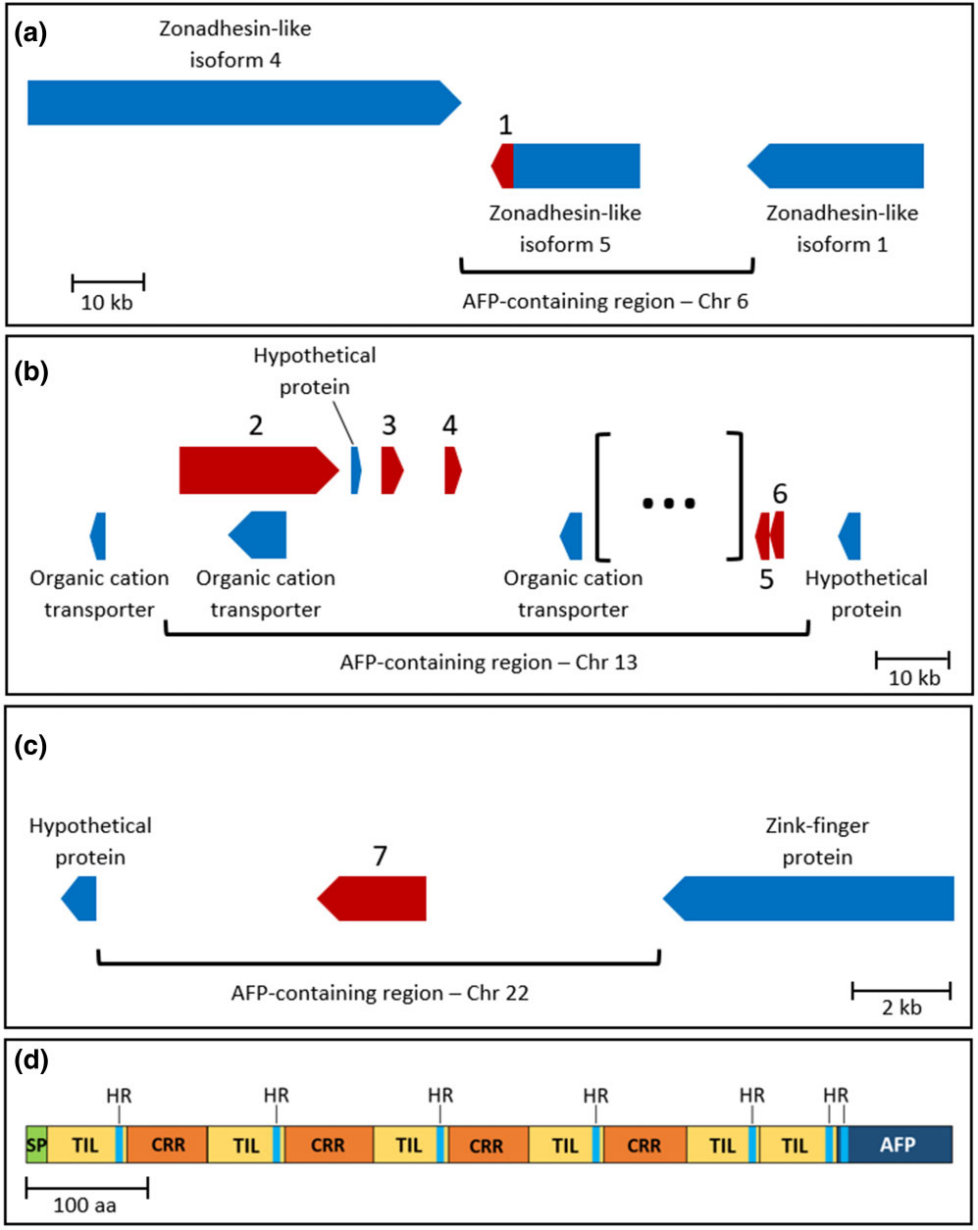
AFP-containing regions in the genome of *N. uddmanniana*. Genes encoding NuAFPs were found on three different chromosomes, Chr. 6, 13 and 22, none of which are homologous to the chromosomes baring AFP genes in *C. fumiferana*. (*a*) The AFP-containing region on Chr. 6 contains a single AFP-coding sequence (NuAFP-1), which BRAKER2 annotation identified as the terminal exon (red) of a “zonadhesin-like” gene (see supplementary file 6, Supplementary Material online, alignment 5), itself flanked by two other “zonadhesin-like” genes. (*b*) The AFP-containing region on Chr. 13 contains five additional AFP genes (NuAFP-2 to -6; red arrows), interspersed with other small genes, several of which encode organic cation transporters (blue arrows). The portion shown as “[…]” represents a 235 kb fragment containing 25 small genes (identified by BRAKER2 annotation), none of which are related to AFPs. (*c*) The AFP-containing region on Chr. 22 contains a single AFP gene (NuAFP-7; red arrow), flanked by two unrelated genes (blue arrows). Loci are drawn to scale, with each arrow diagram representing both exons and introns of a gene (Note: NuAFP-2 is predicted to have a very long intron). Arrows pointing in different directions represent genes found on opposite DNA strands. (*d*) Diagram showing the organization of the zonadhesin-like/AFP protein found on Chr. 6. SP: signal peptide; TIL: TIL cysteine rich domain (see supplementary file 6, Supplementary Material online, alignment 1); CRR: cysteine-rich region (see supplementary file 6, Supplementary Material online, alignment 2); AFP: NuAFP-1; HR: hexapeptide imperfect repeat ([AS][NT]GTC[VI]).

We identified seven homologs of CfAFPs in the genome of *N. uddmanniana*, distributed among three different regions on chromosomes 6, 13, and 22, respectively (see supplementary file 5, Supplementary Material online). Surprisingly, none of these chromosomes were found to be homologous to those containing AFPs in *C. fumiferana* (see supplementary fig. S3, Supplementary Material online), and the regions containing them displayed a distinct organization (fig. 5). Whereas AFP-containing regions on chromosomes 6 and 22 each contained a single AFP (NuAFP-1 and NuAFP-7, respectively; fig. 5*A,C*), the AFP-containing region on chromosome 13 featured five AFP-coding sequences interspersed with other genes, including several “organic cation transporters”. While three of these genes (NuAFP-2, -3, -4) had the same two-exon structure that characterize CfAFPs, the remaining two (NuAFP-5, -6) were present in tandem and lacked the exon encoding a signal peptide (fig. 5*B*).

The most interesting finding was the identification of a single AFP-coding sequence (NuAFP-1) on chromosome 6 that was annotated as the last exon of a putative “zonadhesin-like” protein (fig. 5*A*,*D*; see supplementary file 6, Supplementary Material online), a member of a ubiquitous family of proteins that feature cysteine-rich domains (Hardy and Garbers 1995; Gao and Garbers 1998). The protein identified here is significantly smaller than the large, multi-domain zonadhesins found in vertebrates, but it contains several trypsin inhibitor-like (TIL) cysteine-rich domains (fig. 5*D*; see supplementary file 6, Supplementary Material online, alignment 1), which are found as part of the larger D domains in various proteins including zonadhesins and their “von Willebrand Factor” (vWF) homologs (Hardy and Garbers 1995; Yee and Kretz 2014). The ∼60-amino-acid TIL domains are separated by distinct cysteine-rich regions of approximately the same size; together they form partial repeats of ∼120–130 residues (fig. 5*D*), reminiscent of the D3p repeats identified in mouse zonadhesin (see Gao and Garbers 1998; Herlyn and Zischler 2008; see supplementary file 6, Supplementary Material online, alignment 2). Although the NuAFP-1 portion of the protein showed limited similarity to the upstream cysteine-rich repeats (accounting for why blast searches did not identify this family of proteins as related to CfAFPs), it contained a copy of a hexapeptide imperfect repeat present in each upstream TIL domain (fig. 5*D*) as well as in Nu- and CfAFPs (see supplementary file 6, Supplementary Material online, alignments 3 and 4), suggesting that the NuAFP-1 portion of this protein evolved from a cysteine-rich domain similar to those found upstream of the NuAFP-1 coding sequence.

### *Notocelia* AFPs Display Remarkable Structural Similarity to *Choristoneura* AFPs

**Fig. 6.**
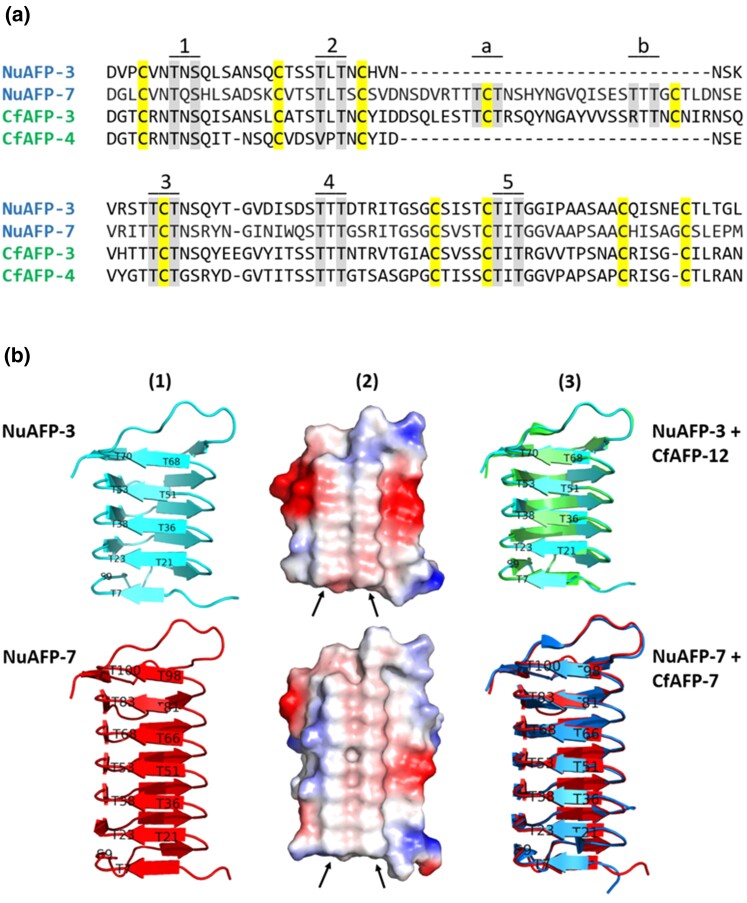
Structural features of *N. uddmanniana* AFPs. (*a*) Manual alignment of a sample of CfAFPs and NuAFPs, featuring both short and long (with 30 aa insert) isoforms. Alignment excludes putative signal peptides. Alignment based on Doucet et al. (2000). Conserved cysteine residues forming disulfide bonds are shaded in yellow, while threonine (or related) residues involved in the five (short isoform) or seven (long isoform) ice binding sites are shaded in grey. The characterized structural properties of CfAFPs are clearly all present in these two NuAFPs. (*b*) Molecular models of NuAFP-3 and NuAFP-7 generated using the AlphaFold 2 structure prediction algorithm and known crystal structures of CfAFPs as templates (PDB ID: 1L0S and 1M8N). (1) Ribbon rendering of NuAFP-3 (short isoform) and NuAFP-7 (long isoform), with labels identifying positions of the paired threonine residues involved in ice binding. (2) Space-filling rendering of the same two AFPs, with arrows pointing to the prominent rows of threonine residues. Surface colors vary from blue (electropositive regions) to red (electronegative regions). (3) Superimposition of each NuAFP model (ribbon rendering) with its CfAFP template (CfAFP-12: green; CfAFP-7: blue), highlighting the structural similarity of the proteins.

In view of the substantial phylogenetic distance separating *Notocelia* from *Choristoneura* (fig. 4), combined with the apparent absence of homologous proteins in three other tortricids showing various degrees of relatedness to the above two species, we asked whether NuAFPs had the structural features known to confer ice-binding activity to CfAFPs. In an amino acid alignment of a sample of NuAFPs and CfAFPs, it was first apparent that *Notocelia* featured both short and long AFP isoforms, suggesting that the emergence of a second isoform type was ancestral to the more recent AFP diversification within the genus *Choristoneura* (fig. 6*A*). In addition, all amino acid residues known to be structurally important in CfAFPs, including cysteine and threonine residues, were clearly present in the two NuAFPs shown in the alignment (fig. 6*A*). Molecular modeling of the same two NuAFPs, based in part on known CfAFP X-ray structures, confirmed the high structural similarity of *Notocelia* and *Choristoneura* AFPs, including the presence of a row of paired threonine residues known to be involved in ice binding (fig. 6*B*). Together, these analyses suggest that NuAFPs (or at least some of them) are likely to display ice-binding activity. Despite this high degree of structural similarity, however, the four NuAFPs that feature a two-exon gene structure like that seen in CfAFPs did not cluster with their presumptive *Choristoneura* orthologs in a ML phylogenetic tree (see supplementary fig. S4, Supplementary Material online).

### Regions Homologous to Those Harboring NuAFPs and CfAFPs in the Other Species Show No Trace of Eroded AFP Coding Sequence

The surprising absence of AFP genes in three tortricid species displaying different degrees of relatedness to *Notocelia* and *Choristoneura* (fig. 4) raised the question of whether, in these species, regions homologous to those featuring AFP genes in *Notocelia* and *Choristoneura* contained traces of eroded AFPs not detected in our original blast searches. To address this issue, we used the genes flanking AFP coding sequences to identify homologous regions in the other species, which were then examined in targeted blastn and tblastn searches. With two possible (and very marginal) exceptions (see supplementary file 7, Supplementary Material online), these analyses revealed no trace of eroded AFP sequences in the homologous sites of the other species. Homologs of the *N. uddmanniana* “zonadhesin-like” protein were found at various locations in all four other tortricid genomes, but all displayed limited similarity to the *N. uddmanniana* polypeptide in the portion corresponding to NuAFP-1 (see example in supplementary file 6, Supplementary Material online, alignment 6). Importantly, none were found within, or in the vicinity of, the CfAFP-containing regions.

### CfAFP Genes Are Expressed Long Before the Advent of Low Temperatures

**Fig. 7.**
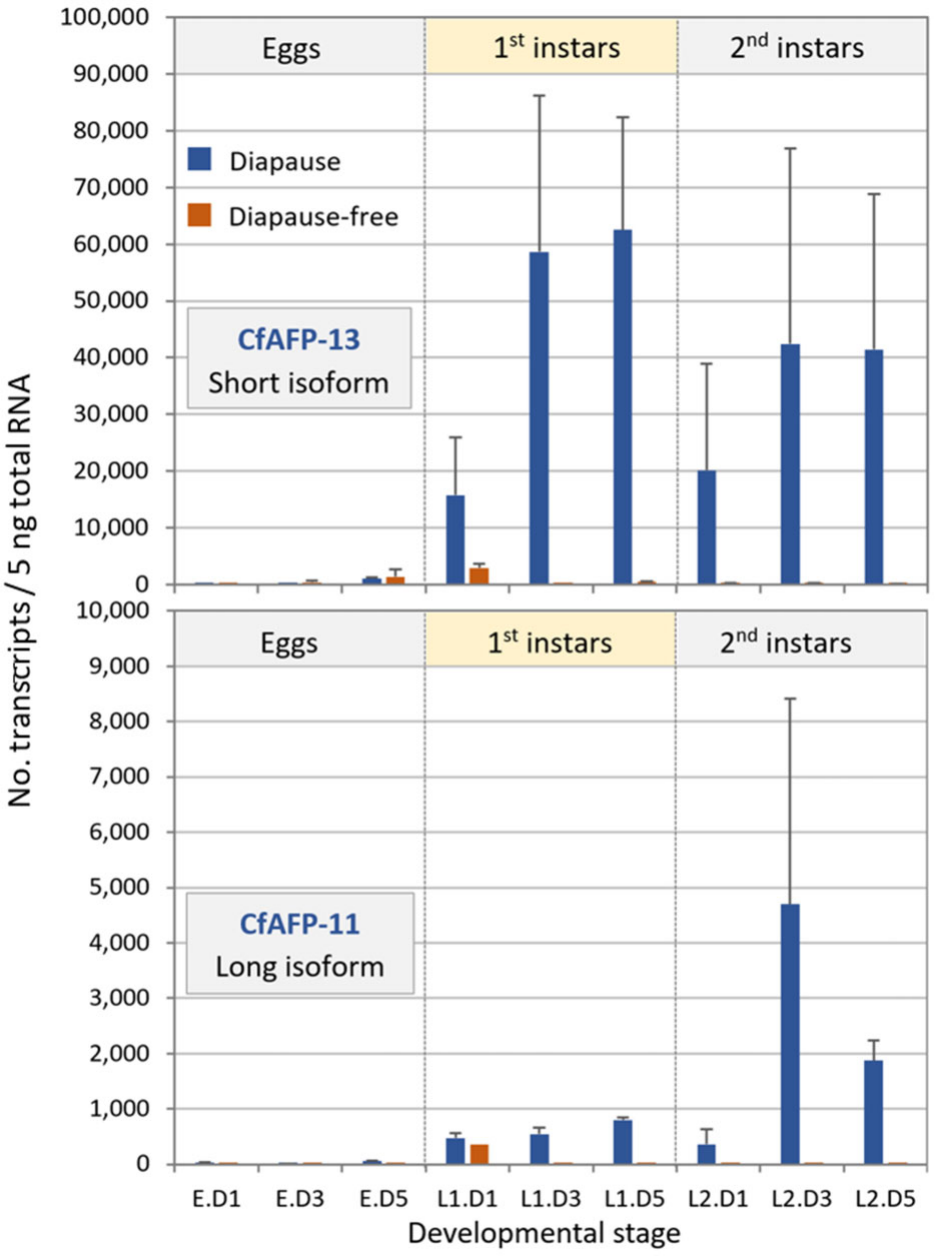
Transcriptional analysis of two CfAFPs in pre- and early diapause *C. fumiferana* larvae. Comparative abundance of CfAFP-13 (short isoform) and CfAFP-11 (long isoform) transcripts in eggs and first and second instar larvae from diapause and diapause-free strains of *C. fumiferana*, as determined by quantitative RT-PCR. Bars show the mean of three biological replicates + SEM. “Dx” in labels on the *x* axis: day of stadium at 22°C.

Previous work, using Northern analyses, showed that some CfAFP genes are expressed during entry into diapause, when temperatures (either experimental or under natural conditions) are still elevated (Doucet et al. 2002; Qin et al. 2007), raising questions about their functions at this early stage of diapause preparation. Here, using a qPCR approach, we confirmed this early expression by comparing transcript abundance of two CfAFPs (a short and a long isoform) between individuals of *C. fumiferana* derived from diapause and diapause-free strains (fig. 7). While CfAFP transcripts were barely detectable in the eggs of both strains, their levels increased sharply in first and early second instars of the diapause strain, whereas they failed to increase appreciably in the corresponding stadia of the diapause-free strain. Interestingly, transcripts of CfAFP-13, a short isoform, were more abundant than those of CfAFP-11, a long isoform (fig. 7), perhaps reflecting lower quantitative requirements for the latter given its higher predicted TH activity.

## Discussion

The origins and convergent evolution of unrelated AFPs in diverse insect and fish taxa are of considerable interest from an evolutionary biology point of view (Cheng 1998; Box et al. 2022). In the present study, we addressed this question in a unique group of AFPs first reported in *C. fumiferana* almost four decades ago (Hew et al. 1983).

Our chromosome-scale assembly of the *C. fumiferana* genome enabled identification of nine previously uncharacterized CfAFPs, including one that may represent a new isoform type (CfAFP-9) and another one believed to be a pseudogene (CfAFP-10). In total, the present assembly features 16 AFP genes, a value that is very close to that estimated earlier based on Southern analysis (∼17; Doucet et al. 2002). This assembly also permitted the characterization of regions harboring AFP genes, which revealed a high concentration of AFP coding sequences within a single site on chromosome 18 (15 of the 16 AFP genes), with some bearing the hallmarks of recent duplication events (figs. 1 and 3). In parallel, comparative genomic analyses identified orthologs of 14 CfAFPs in six congeneric species/subspecies, including taxa that diverged long before *C. fumiferana*, plus seven homologs (NuAFPs) in a tortricid species that belongs to a different subfamily (*N. uddmanniana*: Olethreutinae; *C. fumiferana*: Tortricinae; fig. 4), indicating that CfAFPs are not unique to the genus *Choristoneura* and pointing to the possibility of a phylogenetically early origin of tortricid AFPs.

### Evolutionary Trajectory of Tortricid AFPs

The high degree of structural similarity between NuAFPs and CfAFPs (fig. 6) and the presence of both short and long AFP isoforms in both taxa provide a strong argument in favor of their common origin despite the apparent absence of homologous AFP genes in the three other species considered here. Olethreutinae and the Tortricinae are estimated to have diverged ∼50–75 million years ago (Ma) (Fagua et al. 2017, 2018, 2019). One evolutionary scenario predicts that torticid AFPs arose in an ancestor of these two taxa and selection (e.g. low winter temperature) resulted in their retention in some evolutionarily distant taxa, while they gradually eroded to extinction in taxa not exposed to the same selective pressures. The latter point is suggested by the absence of AFPs in *C. pomonella, A. turbidana*, and *A. honmai*, although it is not clear to what extent these species have evolved under conditions that would make AFPs irrelevant or redundant (e.g. able to avoid freezing with glycerol alone). The current distribution of *A. honmai* is limited to Japan and Korea (Byun et al. 2012), where winter conditions are generally mild, whereas both *C. pomonella* (Pajač et al. 2011) and *A. turbidana* (GBIF Secretariat) have broad geographic ranges that include regions experiencing mild and harsh winter temperatures, respectively. Level of exposure to low temperatures can also depend on whether the insect spends the winter protected (e.g. in the ground) or fully exposed (e.g. on tree branches). For example, larvae of *C. pomonella* overwinter in the litter layer near the base of trees, and low temperatures during the winter season are not considered to represent a major threat to their survival, at least in Central Europe (Rozsypal et al. 2013). Like the two above species, *N. uddmanniana* has a fairly broad latitudinal distribution (GBIF Secretariat), but it overwinters as a third-instar larva between the base of a leaf and the stem of a *Rubus* host, well exposed to winter conditions in some parts of its range (see Meijerman and Ulenberg 2000), where AFPs could improve survival during diapause.

We cannot completely dismiss the possibility that our inability to detect AFPs in the three above species stems from a lack of completeness of their genome assemblies. However, completeness statistics were good for all of them (BUSCO scores ≥ 95%; see supplementary file 4, Supplementary Material online), with the assembly for *A. turbidana*, along with that for *N. uddmanniana*, featuring 28 chromosome-scale scaffolds (= expected number of chromosomes for these two olethreutine species; Šíchová et al. 2013) and BUSCO scores of ∼98%. The fact that chromosomes of *A. turbidana* and *N. uddmanniana* display clear homologous relationships (see supplementary file 1, Supplementary Material online), combined with the observation that sites homologous to the three NuAFP-containing regions were found in *A. turbidana*, but seemed free of any trace of AFP genes (see supplementary file 7, Supplementary Material online), strongly suggests that the absence of AFPs in *A. turbidana* is not artifactual. Similar scenarios likely also apply to *C. pomonella* and *A. honmai*.

Although convergent evolution has been proposed to explain the presence of similar AFPs in distantly related fishes (Chen et al. 1997b; Graham et al. 2013; Zhuang et al. 2019) and intertidal invertebrates (Box et al. 2022), the very high degree of similarity observed here between Cf- and NuAFPs (fig. 6) seems to argue against this hypothesis. However, HGT between *Notocelia* and *Choristoneura* could explain the location of AFP-containing regions on non-homologous chromosomes and the apparent absence of AFP genes in *C. pomonella*, *A. turbidana*, and *A. honmai*. HGT has recently been proposed as a potential explanation for the presence of very similar AFPs in two distantly related coleopteran taxa (Arai et al. 2021). In insects, virus-mediated gene transfer between species has been well documented (Gilbert et al. 2016), sometimes involving portions of DNA large enough to harbor genes such as those encoding AFPs (see Drezen et al. 2017; Zakharov 2016). With such HGTs, however, one would also expect significant intronic similarity between AFP genes in the two species, particularly if the transfer were relatively recent (Graham et al. 2012), which does not seem to be the case here (i.e. introns are highly divergent; data not shown). The directionality of a potential HGT would also need to be assessed, as both *Notocelia* and *Choristoneura* feature homologs of the short and long AFP isoforms, from which duplication and diversification could have occurred independently and at different rates in each taxon. Interestingly, given that the Palearctic distributions of *N. uddmanniana* and *C. murinana* show considerable overlap, an HGT between these two species or between their congeneric ancestors is geographically plausible. Clearly, determining which of the two above scenarios (i.e. vertical descent or HGT) accounts for the presence of homologous AFPs in *Notocelia* and *Choristoneura* will require additional analyses; these include the sequencing of other tortricid genomes, with a focus on freeze-intolerant species that must withstand very low temperatures during the winter and *Choristoneura* species that are not exposed to extreme winter temperatures, in which case AFP gene erosion is expected.

### AFP Diversification within the Genus *Choristoneura*

The presence of orthologous copies of 14 of 16 CfAFP genes in the two phylogenetically most distant *Choristoneura* species examined here (*C. conflictana* and *C. murinana*) indicates that AFP genes had already undergone extensive diversification prior to the speciation event that gave rise to *C. fumiferana*, presumably in response to harsh winter conditions across the genus’ Holarctic range at different periods of its evolutionary history. AFP diversification in the more distantly related species may even have been greater than that reported here for *C. fumiferana*, but the method we used to identify AFP genes (mapping of sequencing reads onto the *C. fumiferana* genome) precluded identification of potentially new AFP paralogs in these genomes. Fish AFPs also tend to form large multigene families where most copies are clustered, sometimes in tandem repeats, as observed here in *C. fumiferana.* Such gene amplification is considered to be a more efficient method of dealing with rapid environmental changes than point mutations that improve activity (Graham et al. 2012). For the AFPs we did identify, the substantial phylogenetic distances that separate *C. fumiferana* from *C. conflictana* and *C. murinana* (fig. 2) make it unsurprising that our read mapping approach did not always recover full AFP genes for these more distant species (see supplementary file 3, Supplementary Material online). Partial gene recovery in these two taxa could also have been caused by genetic drift in AFP sequences, as a consequence of reduced selective pressure in portions of their contemporary ranges (*C. conflictana*: Lumley 2004; *C. murinana*: GBIF Secretariat). Indeed, the possibility that such drift may be taking place in populations less exposed to extreme winter conditions is suggested by the high number of amino acid substitutions among corresponding AFPs in three latitudinally distinct *C. occidentalis* populations. In contrast, *C. fumiferana* homologous AFP pairs were identical in western and eastern populations, exposed to similar winter conditions (see supplementary file 3, Supplementary Material online; fig. 2).

### Ultimate Origin of Tortricid AFPs

The presence of a gene encoding a zonadhesin/AFP polypeptide in the genome of *N. uddmanniana* (fig. 5; see supplementary file 6, Supplementary Material online) provides an argument in favor of tortricid AFPs having evolved through neofunctionalization of an existing protein. Zonadhesins are sperm-specific membrane proteins that owe their name to the role they play in binding to the oocyte’s zona pellucida (egg coat), a glycoprotein. Zonadhesins are large, multi-domain proteins whose D-domains have been found in functionally diverse extracellular proteins including an insect humoral lectin (Gao and Garbers 1998; Kotani et al. 1995). It follows that identification of the *N. uddmanniana* protein as a “zonadhesin-like” polypeptide does not preclude roles unrelated to that attributed to zonadhesins (i.e. adhesion of sperm to oocytes). To our knowledge, no insect zonadhesin has been characterized to date and the AFP-containing protein described here is much smaller than zonadhesins identified so far in vertebrates (752 *vs* 2447 a.a. for pig zonadhesin; Hardy and Garbers 1995), suggesting that the name “zonadhesin-like” may be misleading with respect to the protein’s original function and could be a carry-over from early insect genome annotation efforts.

An affinity for carbohydrates could well have predisposed *N. uddmanniana*’s “zonadhesin-like” protein for the development of ice-binding capacity. Indeed, it has been postulated that the evolution of AFPs from C-type lectins and SAS in fishes and polysaccharide-hydrolyzing molecules in plants may stem from their ability to bind or interact with carbohydrates, as the disposition of OH groups on sugars could resemble ranks of O atoms on some planes of ice (Cheng 1998; Baardsnen and Davies 2001). In this context, the presence of vWF D-domains (and their TIL sub-domains) in the zonadhesin/AFP protein reported here (fig. 5*D*; see supplementary file 6, Supplementary Material online) gives further support to the suggestion that this protein (or a homolog thereof) may have been the progenitor of tortricid AFPs; indeed, these domains are believed to interact with the carbohydrate moieties of their binding targets (Hardy and Garbers 1995; see also Macedo et al. 2004). Future work should focus on determining whether this protein is actually expressed in diapausing *N. uddmanniana* larvae. If expressed, the production of the recombinant polypeptide and an assessment of its ice-binding capacity are needed. If the zonadhesin/AFP protein is indeed the precursor of tortricid AFPs, we surmise that a gene duplication event may have led to the deletion of the zonadhesin-like portion of the gene in one duplicate, followed by the acquisition of a signal peptide, as proposed for type-III fish AFPs (Deng et al. 2010).

### Do Tortricid AFPs Have Functions Other Than Ice-Binding and Could These Have Played a Role in Their Evolution?

In line with earlier observations based on Northern analyses (Doucet et al. 2002; Qin et al. 2007), the qPCR transcriptional data presented here (fig. 7) show that CfAFP expression begins at the onset of the first instar, several weeks before the advent of cold conditions. Although transcripts of the two isoforms examined here were detectable in larvae of the diapause-free strain, their levels were considerably lower than in larvae of the diapause strain, confirming earlier findings that CfAFP transcription is not triggered by changes in environmental conditions, but is developmentally regulated as part of the diapause physiological program (Qin et al. 2007). Why CfAFP gene expression needs to begin so early in the season, at a time when the insect is unlikely to encounter freezing temperatures, is unclear, but the most parsimonious explanation is that accumulation of AFPs must start early to ensure effective freeze protection later in the season, when gene expression may be inhibited under low sub-zero temperatures. Alternative roles for insect AFPs have been proposed, including inhibition of the growth of nonice crystals such as those forming in the primary urine or in the hemolymph (trehalose crystals) of overwintering insects. Some AFPs are also believed to play roles in high temperature tolerance and desiccation resistance (reviewed in Duman and Newton 2020). Of these alternative functions, the latter may seem of relevance to the spruce budworm given that its diapausing second instars display enhanced desiccation resistance relative to post-diapause larvae and pre-diapause first instars (Bauce and Han 2001). However, even though they produce and accumulate AFPs (fig. 7; Qin et al. 2007), pre-diapause first instars undergo a significant decline in their water content, which coincides with a drop of their supercooling point (Han and Bauce 1993), suggesting that dehydration resistance is unlikely to be an important function of CfAFPs. In sum, available evidence suggests that it is the ice-binding capacity of these proteins that drove their evolution, as opposed to some other secondary function.

## Conclusion

The *C. fumiferana* genome assembly reported here enabled a first exploration of the evolutionary history of budworm AFPs, using a comparative genomics approach. These analyses provided evidence for an early diversification of this protein family within the genus *Choristoneura* and revealed the presence of homologous proteins in a distant tortricid species belonging to the genus *Notocelia*. In the latter, we identified a gene whose product, a zonadhesin-related protein, may represent the ancestral condition from which tortricid AFPs evolved through neofunctionalization. Much work remains to be done to clarify the AFP evolutionary path between *Notocelia* and *Choristoneura* and to confirm the role of the zonadhesin-like protein as the precursor of tortricid AFPs. But beyond its usefulness for the analyses reported here, the present chromosome-scale assembly will be a rich resource for a wide range of investigations that address fundamental questions about spruce budworm biology or the design of novel control tools targeting this formidable pest.

## Materials and Methods

### DNA Extraction and Sequencing

DNA was extracted from *C. fumiferana* male pupae obtained from Insect Production and Quarantine Laboratories (IPQL; Great Lakes Forestry Centre, Sault Ste. Marie, ON, Canada), where a colony was established using field material collected in Ontario (Roe et al. 2018). Extractions were carried out on a pool of 30 individuals for PacBio sequencing, whereas we used DNA obtained from a single specimen for Illumina sequencing. DNA was extracted using the Qiagen Blood & Cell Culture DNA midi Kit, following the “Tissue Samples” protocol. Whole insects frozen in liquid nitrogen were ground to a fine powder in a microtube using a pestle. After resuspension in buffer G (containing RNase A and proteinase K) and incubation at 50°C, the sample was centrifuged at 5000 ***g*** for 10 min at 4°C, and the supernatant was transferred onto the Qiagen Genomic-tip; the manufacturer’s protocol was followed for the rest of the extraction procedure, generating yields of 20 and 550 μg for 1 and 30 pupae, respectively.

The bulk of the sequencing conducted for this work was done at the McGill University—Genome Quebec Innovation Centre (Montreal, Canada). Single Molecule, Real-Time (SMRT) sequencing, generating continuous long reads, was performed on a Pacific BioSciences RSII instrument using 15 μg of ∼50 kb DNA fragments previously size-selected by pulse-field gel electrophoresis. Processing of 40 SMRT cells generated 4,622,790 reads (32.04 Gb), corresponding to a raw genome coverage of 62x, based on an estimated genome size of 516 Mb (see Results section). Paired-end, 100-bp sequencing of DNA extracted from a single male pupa was conducted on an Illumina HiSeq2000 instrument, generating 511,563,352 reads (55.25 Gb), which corresponds to a genome coverage of 107x. Additional Illumina sequencing was subsequently performed on a HiSeqX instrument at the Michael Smith’s Genome Sciences Centre (Vancouver, Canada) using DNA extracted from a single male pupa; this sequencing effort generated 1,543,813,666 reads (233.16 Gb), representing a genome coverage of 451x. Both sets of Illumina reads were used for genome polishing purposes only.

### Genome Assembly

Raw PacBio reads were first extracted from the “.bas.h5” files using bash5tools (https://github.com/PacificBiosciences/pbh5tools/). Genome assembly was performed using Canu v1.7 (Koren et al. 2017), with the error rate set as follows: *corOutCoverage* = 200 and -*correctedErrorRate* = 0.15. This “haplotype smashing” approach finds overlaps that are subsequently used for removal of diploidy-related duplications (Phillippy et al. 2020). We initially conducted multiple assemblies, varying the genome size parameter value from 450 to 700 Mb and examining its impact on BUSCO scores (v.5.1.3; Simão et al. 2015); we found that the parameter –*genomeSize* = 640M gave the best BUSCO results. The Canu assembly was submitted to a first round of polishing using the first set of Illumina reads. The latter were mapped onto the assembly using the Burrows-Wheeler Aligner (Li and Durbin 2009), after which Pilon v. 1.22 (https://github.com/broadinstitute/pilon) was run, with the ploidy parameter set to diploid.

At this stage of the assembly process, we observed a high proportion of duplicated BUSCOs (31.8%) presumed to be the outcome of regional duplications of allelic variants, a phenomenon that arises when assembling highly heterozygous genomes (here likely caused by the pooling of 30 individuals for DNA extraction). In an effort to reduce the proportion of such duplications, we ran the “Purge Haplotigs” pipeline (v. 25MAR2018) developed by Roach et al. (2018). This involved the mapping of PacBio reads onto the Pilon-polished assembly, followed by a process of repeat annotation to identify allelic variants and purge duplications. Purge Haplotigs processing reduced the proportion of duplicated BUSCOs to 7.4% with a limited impact on the overall BUSCO scores (changes in assembly metrics along the assembly steps are shown in table 1). The resulting assembly was then submitted to a first scaffolding step with SSPACE v. 1.1 (Boetzer et al. 2011), using the raw PacBio reads.

Further scaffolding was carried out by a service provider (Dovetail Genomics, Scott’s Valley, CA, USA), using our SSPACE assembly as input scaffolds. Chicago® and Dovetail™ Hi-C libraries were constructed using high-molecular-weight DNA extracted from a single male pupa and sequenced on an Illumina HiSeqX instrument. These scaffolding technologies use proximity ligation and massively parallel sequencing to probe the three-dimensional structure of chromosomes within the nucleus and capture interactions by paired-end Illumina sequencing (Burton et al. 2013; Kaplan and Dekker 2013; Marie-Nelly et al. 2014). The final assembly was generated using the Dovetail™ HiRise pipeline, which produced 30 chromosome-length scaffolds, plus 304 smaller scaffolds. We used BUSCO analysis to compare the completeness of the 30-scaffold assembly with that comprising all 334 scaffolds; since inclusion of the smaller scaffolds only led to an increase in the proportion of duplicated BUSCOs, all downstream work was conducted on the 30 chromosome-length scaffolds only, including annotation. A final Pilon polishing step was carried out as described above, but using the second set of Illumina reads instead. This polishing step resulted in 3,231,146 corrections being made to the HiRise assembly (table 1).

### Genome Annotation

Prior to launching the MAKER annotation pipeline V. 3.01.03 (Cantarel et al. 2008), RepeatModeler V. 2.0.1 (Flynn et al. 2020) was run to identify repeated elements within the spruce budworm genome. In round 1 of MAKER annotation, repeats were first masked based on the RepeatModeler output, and a first gene model construction step was carried out as guided by the *C. fumiferana* transcriptome (see NCBI BioProject PRJNA750049 and see supplementary file 8, Supplementary Material online) and all lepidopteran proteins that could be retrieved from GenBank. Round 1 generated a set of GFF files that were used to train the Augustus V. 3.3.3 (Stanke et al. 2008) and SNAP V. 2006-07-28 (Korf 2004) algorithms, the outputs of which were then used in round 2. The latter was launched with a view to refining the round-1 gene models, using both *ab initio* gene prediction algorithms and the Augustus and SNAP data generated at the end of round 1. The refined gene models produced by round 2 were used again to train the Augustus and SNAP algorithms; their outputs were employed for the final (round 3) MAKER gene model refinement step. The data generated by round 3 were then used to conduct functional annotation of each gene model through homolog searches against the InterProScan V. 5 database (Jones et al. 2014). Subsequent editing of automated annotation was conducted for genes of interest to our group, including those featured in the present paper, using Apollo v.2.6.4. (Dunn et al. 2019).

### Genome Size Assessment

Size of the *C. fumiferana* genome was assessed using flow cytometry of propidium iodide-stained nuclei, as described by Hare and Johnston (2011). Nuclei were isolated from both male (*N* = 5) and female (*N* = 4) *C. fumiferana* adult heads (moths obtained from IPQL, Great Lake Forestry Centre, Sault Ste.Marie, Canada) and shipped on dry ice to the laboratory of J.S. Johnston (Texas A&M University, College Station, TX, USA). Analyses were performed on a Partec-CyFlow® flow cytometer, using nuclei of single individuals co-prepared with nuclei from a *Drosophila virilis* female standard (1C = 328 Mbp).

### Comparative Genomic Analyses

Proteins encoded by the *C. fumiferana* genome were compared with those found in three other lepidopteran genomes (*Plutella xylostella* [Plutella_xylostella_DBM_FJ_v1.1_-_proteins.fa; http://download.lepbase.org/v4/sequence/], *Bombyx mori* [https://www.uniprot.org/proteomes/UP000005204], and *Papilio machaon* [https://www.uniprot.org/proteomes/UP000053240]) using OrthoMCL (Li et al. 2003), as well as in-house scripts developed to generate separate fasta files for orthologous groups with specific features (see https://github.com/Patg13/OrthoMCL_Results_Processing).

To infer evolutionary history, we searched for orthologs of all CfAFPs in other *Choristoneura* species (*C. occidentalis occidentalis*, *C. occidentalis biennis, C. pinus, C. rosaceana, C. conflictana, C. murinana*) first by mapping Illumina reads (obtained for these species in the context of an earlier study; Fagua et al. 2018) onto the *C. fumiferana* genome and then by extracting the relevant coding sequences from the regions where AFPs were identified in the reference genome (see Results for details). The *Choristoneura* AFPs we obtained were used in phylogenetic analyses (see below).

Publicly available tortricid genomes (as of 2021-07-07; GCA_905163555.1 [*Notocelia uddmanniana*], GCA_905147355.2 [*Apotomis turbidana*], GCA_003425675.2 [*Cydia pomonella*], and GCA_005406045.1 [*Adoxophyes honmai*]) were searched for homologs of *Choristoneura* AFPs using the latter as queries in local tblastn searches performed in BioEdit (Hall 1999). The coding sequences of candidate AFP homologs were deduced using the gene structure of characterized CfAFPs (Doucet et al. 2002) as guide. Tortricid genomes in which AFPs were not found using the above approach were further scrutinized with a view to detecting incipient or eroded sequences signaling either the birth or disappearance of AFP genes. To this end, genomes were searched for homologs of the *C. fumiferana* and *N. uddmanniana* AFP-containing regions, first using the MAKER annotation output to identify PCGs flanking each of the two AFP-containing regions in *C. fumiferana*; these proteins were then used as queries in local tblastn searches against the other tortricid genomes to identify homologous regions, an approach made possible by the high degree of synteny in the Lepidoptera (Pringle et al. 2007). These regions were then examined more closely for the presence of faint AFP-like signal using CfAFP exons and introns as queries in blastn searches as well as AFP amino acid sequences in tblastn searches. Since no *N. uddmanniana* genome annotation was available at the time of conducting the present work, searches for homologs of NuAFP-containing regions in other tortricid genomes first required that we obtain an annotation of the three chromosomes bearing AFP genes in this species (Chr. 6, 13 and 22). To this end, we employed the BRAKER2 annotation pipeline (Brůna et al. 2021), which does not require inclusion of transcriptomic data as extrinsic evidence. Once PCGs flanking the NuAFP-containing regions were identified, analyses were carried out as described above for CfAFP-containing regions.

### Phylogenetic Analyses

A phylogenetic tree was constructed for the five tortricid species whose genomes were searched for AFPs using the ML method and the Tamura-Nei model (Tamura and Nei 1993), as implemented in MEGA X (Kumar et al. 2018). For tree construction, we used the concatenated nucleotide sequences encoding the 13 mitochondrial PCGs of all five species. For some species, the sequence of a given PCG could not be fully recovered from Genbank; as a result, all other aligned sequences were trimmed to retain only sites with no missing data.

With respect to AFPs, we conducted a series of phylogenetic analyses using nucleotide and amino acid sequences under ML and Bayesian inference approaches implemented in IQ-TREE v. 1.6.12 available on the web server (Nguyen et al. 2015; Trifinopoulos et al. 2016) and MrBayes v.3.2.7 (Ronquist et al. 2012). A detailed description of the analyses we conducted is provided in supplementary file 9, Supplementary Material online.

### Synteny/Chromosome Homology Analysis

To determine whether *C. fumiferana* and *N. uddmaniana* AFP-containing regions were on homologous chromosomes, the entire *C. fumiferana* proteome (i.e. output of MAKER annotation) was mapped onto the genome of *N. uddmanniana* (GenBank ID: 98138) using tblastn as implemented in BioEdit (Hall 1999). Only proteins displaying a unique hit on the *N. uddmanniana* genome, with an *e*-value ≤ 1.0 e-20, were retained for the analysis. A graphical representation of the synteny results was generated using the R package *circlize* (Gu et al. 2014). A similar approach was used to identify homologous chromosomes in *A. turbidana* (GCA_905147355.2) but using only a random sample of ∼50 proteins per *C. fumiferana* chromosome as queries in the tblastn searches.

### Molecular Modeling

Molecular models of two *N. uddmanniana* AFPs (NuAFP-3 and NuAFP-7) were generated using the AlphaFold 2 structure prediction algorithim and known crystal structures of CfAFPs as templates (PDB ID: 1L0S and 1M8N). AlphaFold 2 is a machine learning approach that incorporates information extracted from amino acid sequences, multiple sequence alignments and homologous protein structures (Jumper *et al*. 2021). Images and structure alignments were generated using PyMOL (http://www.pymol.org).

### qPCR Assessment of Gene Expression

Total RNA was extracted on days 1, 3, and 5 of each of three *C. fumiferana* life stages, namely eggs, first instars (“L1s”), and second instars (“L2s”), obtained from both the standard IPQL diapause strain and from one that was selected (Harvey 1957) to skip diapause (“diapause-free”). For each sampling point, insects (eggs: ∼65 mg/sample; L1s: ∼40 insects/sample; L2s: ∼10 insects/sample) reared at 22°C (16 h light: 8 h dark, 55% RH) were ground in TRIzol reagent (Thermo Fisher Scientific) and RNA was extracted using the Direct-zol RNA mini-prep kit (Zymo Research) with the facultative DNase step, following the manufacturer’s protocol. RNA was quantified using a NanoDrop 1000 spectrophotometer (Thermo Fisher Scientific). cDNA was synthesized from 1 µg total RNA, using the QuantiTect Reverse Transcription kit (Qiagen), following the manufacturer’s instructions. At the end of the procedure, samples were brought to a concentration equivalent to 5 ng total RNA/µL through dilution with 10 mM Tris-HCl, pH 8. Primers (see supplementary table 2, Supplementary Material online) were designed using the OligoAnalyzer™ Tool (https://www.idtdna.com/pages/tools/oligoanalyzer?returnurl=%2Fcalc%2Fanalyzer). Criteria for primer selection included a Tm of 60°C and predicted amplicon size ranging between 100 and 200 bp. Quantitative PCR was performed using an Applied Biosystems 7500 Fast Real-Time PCR machine. Ninety-six-well BrightWhite real-time PCR plates (Primerdesign, UK) were used with Applied Biosystems MicroAmp Optical Adhesive Film. Fifty PCR cycles of 95^°^C, 15 s; 60°C, 30 s; and 65°C, 90 s were performed using the Qiagen Quantitect SYBR Green PCR Kit. Three technical replicates were amplified for each of three biological samples. Quantification was done using 1 µL of cDNA reaction per well (i.e. 5 ng total RNA equivalent). To confirm the specificity of reaction, a melting curve analysis was carried out at the end of the PCR run. Linear regression of efficiency analysis developed for modeling qPCR amplification was used to determine absolute quantities of target molecules (Rutledge 2011). Lambda genomic DNA was used as a quantitative standard.

## Supplementary Material

Supplementary data are available at *Genome Biology and Evolution* online.

## Supplementary Material

evac087_Supplementary_Data

## Data Availability

The data underlying this article are available at the NCBI under BioProject accession numbers PRJNA737804 (genome) and PRJNA750049 (transcriptome).
