# Supplementary material for: The Spruce Budworm Genome: Reconstructing the Evolutionary History of Antifreeze Proteins
Source: Genome Biol Evol. 2022 Jun 7;14(6):evac087. doi: 10.1093/gbe/evac087 (PMC9210311; doi:10.1093/gbe/evac087)
Supplement: evac087_Supplementary_Data [file evac087_supplementary_data.zip › Supplementary figures_rev.pdf]

Supplementary figures for the manuscript entitled:

The Spruce Budworm Genome: Reconstructing the Evolutionary History of Antifreeze Proteins

by

Béliveau C, Gagné P, Picq S, Vernygora O, Keeling CI, Pinkney K, Doucet D, Wen F, Johnston JS, Maaroufi H, Boyle B, Laroche J, Dewar K, Juretic N, Blackburn G, Nisole A, Brunet B, Brandao M, Lumley L, Duan J, Quan G, Lucarotti CJ, Roe AD, Sperling FAH, Levesque RC, Cusson M.

Genome Biology and Evolution

Supplementary fig. 1: Life cycle of *C. fumiferana*………………………………………………………………….. p. 2

Supplementary fig. 2: Numbers of shared and unique orthologous groups among four lepidopteran species……………………………………………........................... p. 3

Supplementary fig. 3: *C. fumiferana-N. uddmanniana* synteny/chromosome homology analysis………………………………………………………………………………………. p. 4

Supplementary fig. 4: ML phylogeny of four AFP orthologs among *Choristoneura* species and *N. uddmanniana*.……………………………………………………………………………… p. 5

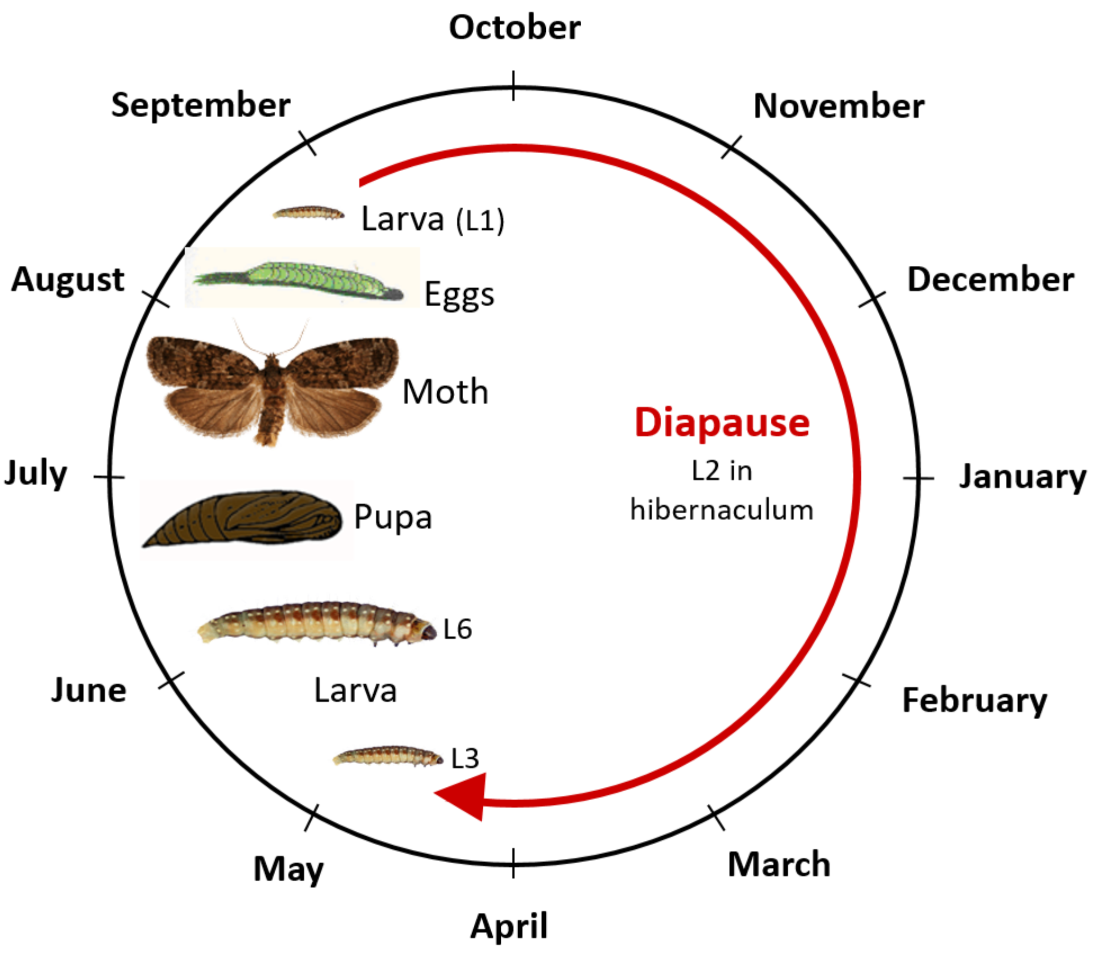

Supplementary Fig. 1. Life cycle of *C. fumiferana*. The spruce budworm is known as a univoltine species that spends more than half of its life as a second instar (L2) in diapause. Under some environmental conditions, however, some larvae will undergo a second diapause as 4th instars, extending the life cycle to two years (see Marshall and Roe 2021).

Marshall KE, Roe AD. 2021. Surviving in a frozen forest: the physiology of eastern spruce budworm overwintering. *Physiology* 36:174-182.

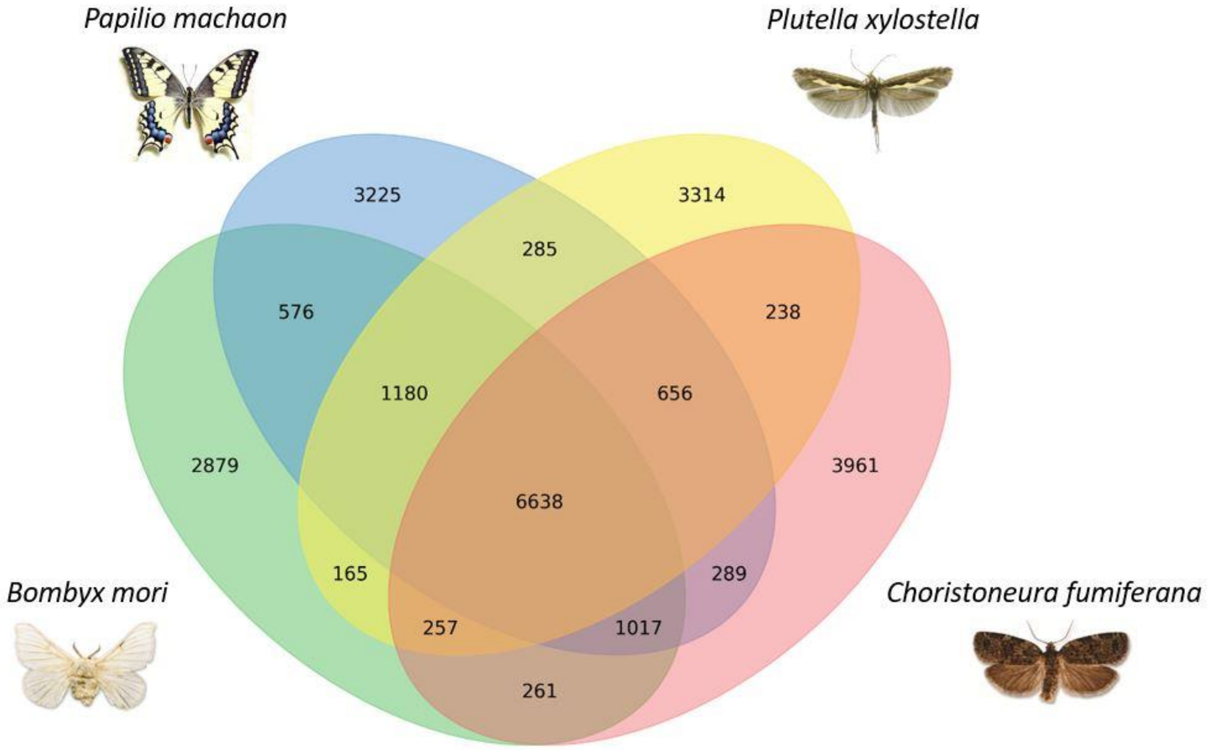

Supplementary Fig. 2. Numbers of shared and unique orthologous groups among four lepidopteran species, including *C. fumiferana, P. xylostella, P. machaon* and *B. mori*, as determined using OrthoMCL (see Materials and methods for details).

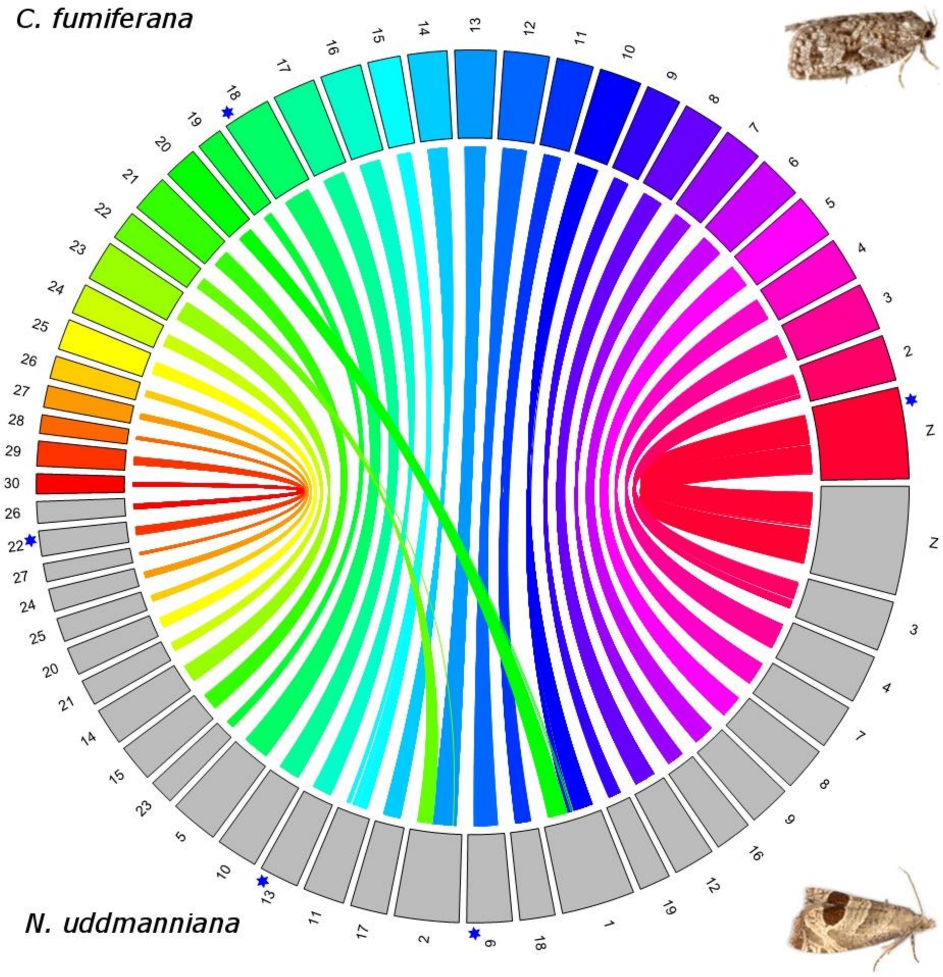

Supplementary Fig. 3. ***C. fumiferana-N. uddmanniana*** **synteny/chromosome homology analysis.** Mapping of *C. fumiferana* proteins onto the *N. uddmanniana* genome. Each colored box represents one of the 30 *C. fumiferana* chromosomes while each grey box represents one of the 28 *N. uddmanniana* chromosomes. Each of the 8067 connector lines identifies a one-to-one orthologous match between *C. fumiferana* and *N. uddmanniana* (tblastn analysis, expected value cut-off: 1.0 e-20). Note that chromosomes 1 and 2 in *N. uddmanniana* correspond to the fusion of *C. fumiferana* chromosomes 10 and 20, and 13 and 22, respectively. Blue stars: chromosomes harboring AFPs.

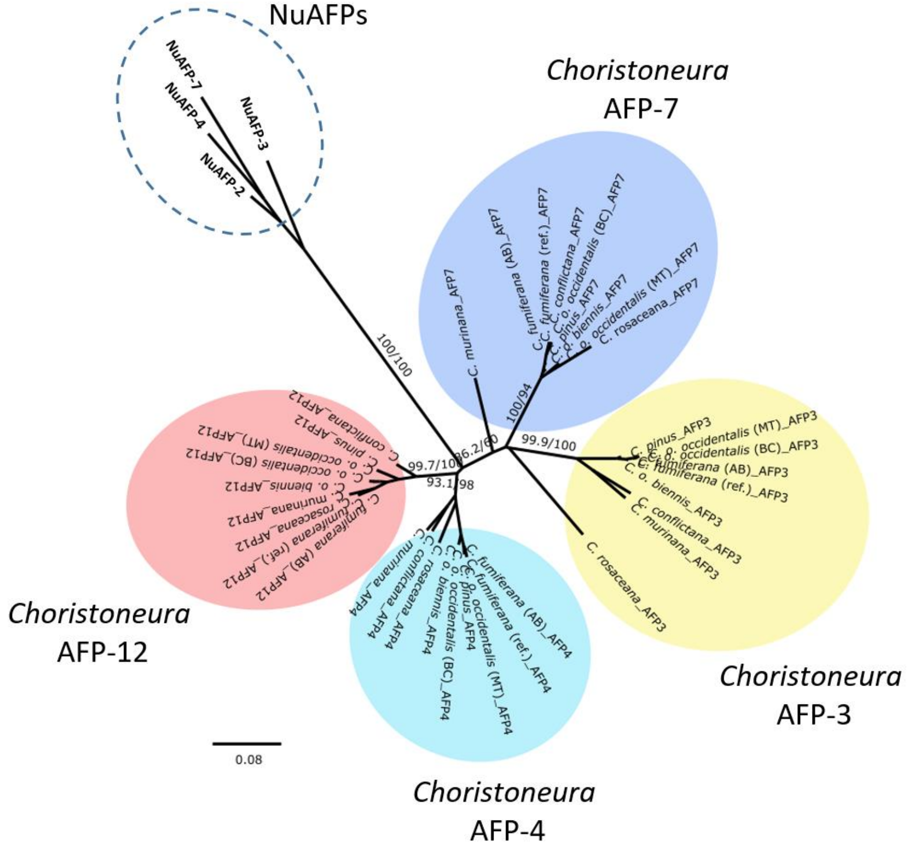

Supplementary Fig. 4. ML phylogeny of four AFP orthologs among *Choristoneura* species and *N. uddmanniana*. Four of the seven AFP genes detected in the genome of *N. uddmanniana* feature a two-exon structure typical of *C. fumiferana* AFPs; these genes display the following putative orthologous relationships: NuAFP-2 → CfAFP-4; NuAFP-3 → CfAFP-12; NuAFP-4 → CfAFP-3; NuAFP-7 → CfAFP-7. Nucleotide sequences of AFPs in these orthologous groups were used to construct a maximum likelihood tree using the IQ-TREE software. Tree topology shows that *N. uddmanniana* AFPs form a distinct cluster, independent of their orthologous relationship with *Choristoneura* AFPs. ML analyses conducted on the amino acid sequences, as well as Bayesian analyses using either amino acids or nucleotides, generated trees with almost identical topologies (see supplementary file 9). Numbers at the nodes indicate SH-aLRT/ultrafast bootstrap support values.
