# Supplementary material for: The Spruce Budworm Genome: Reconstructing the Evolutionary History of Antifreeze Proteins
Source: Genome Biol Evol. 2022 Jun 7;14(6):evac087. doi: 10.1093/gbe/evac087 (PMC9210311; doi:10.1093/gbe/evac087)

Supplementary files 4, 6, 8 and 9 for the manuscript entitled:

The Spruce Budworm Genome: Reconstructing the Evolutionary History of Antifreeze Proteins

by

Béliveau C, Gagné P, Picq S, Vernygora O, Keeling CI, Pinkney K, Doucet D, Wen F, Johnston JS, Maaroufi H, Boyle B, Laroche J, Dewar K, Juretic N, Blackburn G, Nisole A, Brunet B, Brandao M, Lumley L, Duan J, Quan G, Lucarotti CJ, Roe AD, Sperling FAH, Levesque RC, Cusson M.

Genome Biology and Evolution

Supplementary file 4: BUSCO scores for genomes used in comparative genomics analyses……………………………………………………………………………………….. p. 2

Supplementary file 6: "Zonadhesin-like" gene in the AFP locus of *Notocelia uddmanniana* chromosome 6……………………………………………………………………………… p. 4

Supplementary file 8: *Choristoneura fumiferana* transcriptome assembly…………………………..p. 10

Supplementary file 9: Phylogenetic analyses of antifreeze proteins……………………………………p. 11

Supplementary file 4 – BUSCO scores for genomes used in comparative genomics analyses

BUSCO version: 5.1.3
The lineage dataset: lepidoptera_odb10
(Creation date: 2020-08-05, number of genomes: 16, number of BUSCOs: 5286)
BUSCO run in mode: genome
Gene predictor used: metaeuk
Dependencies and versions:
hmmsearch: 3.2; metaeuk: 9dee7a78db0f2a8d6aafe7dbf18ac06bb6e23bf0-MPI

A. Species included in the analysis shown in Figure 4:

1. *Choristoneura fumiferana*

C:97.2%[S:93.3%,D:3.9%],F:0.5%,M:2.3%,n:5286
5140 Complete BUSCOs (C)
4932 Complete and single-copy BUSCOs (S)
208 Complete and duplicated BUSCOs (D)
28 Fragmented BUSCOs (F)
118 Missing BUSCOs (M)
5286 Total BUSCO groups searched

2. *Notocelia uddmanniana* (GenBank: GCA_905163555.1)

C:98.3%[S:97.6%,D:0.7%],F:0.5%,M:1.2%,n:5286
5197 Complete BUSCOs (C)
5159 Complete and single-copy BUSCOs (S)
38 Complete and duplicated BUSCOs (D)
26 Fragmented BUSCOs (F)
63 Missing BUSCOs (M)
5286 Total BUSCO groups searched

3. *Cydia pomonella* (GenBank: GCA_003425675.2)

C:94.8%[S:88.3%,D:6.5%],F:1.2%,M:4.0%,n:5286
5014 Complete BUSCOs (C)
4670 Complete and single-copy BUSCOs (S)
344 Complete and duplicated BUSCOs (D)
66 Fragmented BUSCOs (F)
206 Missing BUSCOs (M)
5286 Total BUSCO groups searched

4. *Apotomis turbidana* (GenBank: GCA_905147355.2)

C:98.2%[S:97.5%,D:0.7%],F:0.5%,M:1.3%,n:5286
5194 Complete BUSCOs (C)
5156 Complete and single-copy BUSCOs (S)
38 Complete and duplicated BUSCOs (D)
25 Fragmented BUSCOs (F)
67 Missing BUSCOs (M)
5286 Total BUSCO groups searched

5. ***Adoxophyes honmai*** (GenBank: GCA_005406045.1)

C:95.2%[S:94.5%,D:0.7%],F:2.3%,M:2.5%,n:5286
5035 Complete BUSCOs (C)
4997 Complete and single-copy BUSCOs (S)
38 Complete and duplicated BUSCOs (D)
122 Fragmented BUSCOs (F)
129 Missing BUSCOs (M)
5286 Total BUSCO groups searched

B. Species other than *C. fumiferana* included in the analysis shown in Supplementary Figure 2:

1. ***Plutella xylostella*** (GenBank: GCF_905116875.1)

C:96.9%[S:95.9%,D:1.0%],F:1.0%,M:2.1%,n:5286
5121 Complete BUSCOs (C)
5069 Complete and single-copy BUSCOs (S)
52 Complete and duplicated BUSCOs (D)
54 Fragmented BUSCOs (F)
111 Missing BUSCOs (M)
5286 Total BUSCO groups searched

2. ***Bombyx mori*** (GenBank: GCA_014905235.2)

C:98.7%[S:97.8%,D:0.9%],F:0.4%,M:0.9%,n:5286
5214 Complete BUSCOs (C)
5169 Complete and single-copy BUSCOs (S)
45 Complete and duplicated BUSCOs (D)
19 Fragmented BUSCOs (F)
53 Missing BUSCOs (M)
5286 Total BUSCO groups searched

3. ***Papilio machaon*** (GenBank: GCA_912999745.1)

C:97.8%[S:97.1%,D:0.7%],F:1.0%,M:1.2%,n:5286
5173 Complete BUSCOs (C)
5135 Complete and single-copy BUSCOs (S)
38 Complete and duplicated BUSCOs (D)
53 Fragmented BUSCOs (F)
60 Missing BUSCOs (M)
5286 Total BUSCO groups searched

Supplementary file 6 – "Zonadhesin-like" gene in the AFP locus of *Notocelia uddmanniana* chromosome 6

BRAKER2 annotation of *Notocelia uddmanniana* chromosome 6 resulted in the identification of a "zonadhesin-like" gene within the region harbouring NuAFP-1. The coding sequence of this gene features 12 exons, where the last one corresponds to NuAFP-1 (without signal peptide). The organization of this gene, its coding sequence and the encoded protein are shown below, followed by six sequence alignments.

Note : this gene is on the (–) strand.

**stop_codon**	**28693504**	**28693506**	
exon - 12	28693504	28693784	***Nu*AFP-1**
intron	28693785	28695461	
exon - 11	28695462	28695650	
intron	28695651	28696329	
exon - 10	28696330	28696518	
intron	28696519	28696930	
exon - 9	28696931	28697113	
intron	28697114	28698726	
exon - 8	28698727	28698927	
intron	28698928	28700261	
exon - 7	28700262	28700441	
intron	28700442	28702153	
exon - 6	28702154	28702351	
intron	28702352	28704241	
exon - 5	28704242	28704442	
intron	28704443	28704820	
exon - 4	28704821	28705021	
intron	28705022	28706151	
exon - 3	28706152	28706334	
intron	28706335	28709102	
exon - 2	28709103	28709300	
intron	28709301	28714107	
exon - 1	28714108	28714162	
**start_codon**	**28714160**	**28714162**	

Complete coding sequence on the (–) strand:

CTAAGCGGCACCCACGATGCATCCAGAGATGACACACGCAGCAGATGGGGCAGGTATCCCTCCAGTAATAGTGCA
AGAGGTAACTGAGCATCCCGGTCCTGTGAGAGTAGTGCCAGTAGTTGTCGAGTCAGTAATATCAACGCCGTTGTA
CTGGCTGTTTGTACAGGTAGTAGTTGTGACGTCACTGTTGTTCACACTACAATCGGTCAGCGTTGACATATTGCACT
GGGAGTCAGCGCTGACATCACTTCTCGTGTTGACACACGTGCCATTCGAAGGGACTGGGCATTGGTCAATTGGCA
CGCAAGTCCCATTCGAAGCTCTCAAGTACCCTTTGATGCAGACACATCCCACGATACAGGTTACAGGATCTACGCA
GATAGTTGGCTGGCCAACCTGGCTGCAATTTCTAAGATCGCATCCGCCATTGATGCAGGTAGATAATTCTTCATTA
ACTTTGCAGATGGCTGGTGGGCATTGGTCAGTTGGCACGCAAGTCCCATTTGAAGCTCTCAAGTACCCTTTGACGC

AGACACATCCCTTGATACAAGTTACAGGATCCACGCATATATTTGGCTGGCCAACTTGGCTGCAATTTTTAGGGCC
GCATCCACCATTGATGCAGggagaatattcttcattaACTTCGCAAATGGGTGTGCAATCCTGTGGCAAGACGCACTTTTT
AACATTGTCATCGAATACAAAGCCGTCCTTGCAGTCGCAGCCGATAGGTTTGCACACTATACGGGGGCAAATTGG
GAATTTAACCTTGTAGGTGCTACAAGTCTTCCCACAGTTGTACCCACATCCTGGCTGTGCATTTGGATCACCGCCAC
ACCCGGTAGGAGGACATTTATCTTGAGGAATACAAGTCCCGTTAGCGGCACGTAGATATCCTGAAGCACAGATGC
ACCCCTTGATGCAAGACTCTGGTGTTATGTCGATACATAACTCAGGCTGGCCAAGTTGGCTGCAGTTTCTGTGGCC
ACATCCTCCGTTTATGCAACTCGAGTATACTTCGTTAAGTCCACATGTGGGCGTGCAATCTTTAGGCAAAACGCATT
TCTTGACATTAGGGTCGAAGACAAAGCCGTCATTGCAATCGCATCCATTGAGGTAGCAGATCAAAATACAGGCtac
atttttgattttgtaagtggAACACGTCCTTCCACAGTTGACGCCACAACCGGGGTGCGAGTTTGGGTCATTACCGCAACCT
TCATTGCACTGATTCTTTGGTACACAAGTTCCGTTCGCGGAGCGCAAGTAGCCTGGTTGGCACACACAGCCTTTAA
TGCAGCTCTTAGGGTCCAGTTTCACGCAAGGCACTGGTTTGCCAGCCTGACTGTAGTTCCGGGCTTCACAGTCCCC
GTTGGTGCAGTTCGAGAACACTTCATTGGGTCCACATTTATAGTGGCATTGATCTCTGGGAACACACTTTTTAGCA
GCGTTCCGTGCGTAACCGTCAATGCACCTACAAGCTGGCTTGCACGTAGGATCGCCGTATTGAGGGCTCGGTGCA
CACAGTACAAGTGCTGGGTTAACATCACAGCTGTCACCGGGGCATGGCGCTGGACATGAGTCGTATATTTCGTTTG
GTCGGTCACAAGTTAGAGGTTGTTCGCATTGCTTCCTGAGCACACATGTGCCCGTAGCGTTTCTCGCGTAGCCGTC
GGCGCACCGGCATCCGGGCTTGCATTGTGGATCCCCTGGCTTCGGCGGCGCGGCGCATTTAATGACTCTGTCATCC
ACATCGCACCGACGCGGCGGGCACGGCGCGGGACAAACATCGTATACCTCGTTTATACCGCAGATGATGCAATCC
TTTGGCAAGACGCACTTTTTAACATTGCCATCGTATACATAGCCATCCTTGCAGTCGCAGCCGTTAGGTTTACAAGG
AATACGGGGGCAAATTGGGAGTAAAGTCTCGTAGGTGGTACAAGTCTTCCCACAGTAGTACCCACATCCTGGTTT
AGCGTTTGGATCACCGCCACACCCTTCATTGCACTGATTCTGTGGTACACAGGTTCCGTTCGCGGAGCGCAAGTAG
CCTGGTTTGCACACACAGCCTTTAATGCAGCTCTTAGGGTCCAGTTTCACGCAAGGCACTGGTTTGCCAGCCTGACT
GCAATTGCGAGGTTCACAGCCCCCGTTGGTGCAGTTCGAGAACACTTCATTGGGTCCACATTTATAGTCATTGCAA
GAGACTATTGCAACCACGCAGGTCAAAAGCACAAATGTCTTCCACAT

Reverse complement of above coding sequence

ATGTGGAAGACATTTGTGCTTTTGACCTGCGTGGTTGCAATAGTCTCTTGCAATGACTATAAATGTGGACCCAATG
AAGTGTTCTCGAACTGCACCAACGGGGGCTGTGAACCTCGCAATTGCAGTCAGGCTGGCAAACCAGTGCCTTGCG
TGAAACTGGACCCTAAGAGCTGCATTAAAGGCTGTGTGTGCAAACCAGGCTACTTGCGCTCCGCGAACGGAACCT
GTGTACCACAGAATCAGTGCAATGAAGGGTGTGGCGGTGATCCAAACGCTAAACCAGGATGTGGGTACTACTGT
GGGAAGACTTGTACCACCTACGAGACTTTACTCCCAATTTGCCCCCGTATTCCTTGTAAACCTAACGGCTGCGACTG
CAAGGATGGCTATGTATACGATGGCAATGTTAAAAAGTGCGTCTTGCCAAAGGATTGCATCATCTGCGGTATAAA
CGAGGTATACGATGTTTGTCCCGCGCCGTGCCCGCCGCGTCGGTGCGATGTGGATGACAGAGTCATTAAATGCGC
CGCGCCGCCGAAGCCAGGGGATCCACAATGCAAGCCCGGATGCCGGTGCGCCGACGGCTACGCGAGAAACGCTA
CGGGCACATGTGTGCTCAGGAAGCAATGCGAACAACCTCTAACTTGTGACCGACCAAACGAAATATACGACTCAT
GTCCAGCGCCATGCCCCGGTGACAGCTGTGATGTTAACCCAGCACTTGTACTGTGTGCACCGAGCCCTCAATACGG
CGATCCTACGTGCAAGCCAGCTTGTAGGTGCATTGACGGTTACGCACGGAACGCTGCTAAAAAGTGTGTTCCCAG
AGATCAATGCCACTATAAATGTGGACCCAATGAAGTGTTCTCGAACTGCACCAACGGGGACTGTGAAGCCCGGAA
CTACAGTCAGGCTGGCAAACCAGTGCCTTGCGTGAAACTGGACCCTAAGAGCTGCATTAAAGGCTGTGTGTGCCA
ACCAGGCTACTTGCGCTCCGCGAACGGAACTTGTGTACCAAAGAATCAGTGCAATGAAGGTTGCGGTAATGACCC
AAACTCGCACCCCGGTTGTGGCGTCAACTGTGGAAGGACGTGTTccacttacaaaatcaaaaatgtaGCCTGTATTTTGAT
CTGCTACCTCAATGGATGCGATTGCAATGACGGCTTTGTCTTCGACCCTAATGTCAAGAAATGCGTTTTGCCTAAAG
ATTGCACGCCCACATGTGGACTTAACGAAGTATACTCGAGTTGCATAAACGGAGGATGTGGCCACAGAAACTGCA
GCCAACTTGGCCAGCCTGAGTTATGTATCGACATAACACCAGAGTCTTGCATCAAGGGGTGCATCTGTGCTTCAGG

ATATCTACGTGCCGCTAACGGGACTTGTATTCCTCAAGATAAATGTCCTCCTACCGGGTGTGGCGGTGATCCAAAT
GCACAGCCAGGATGTGGGTACAACTGTGGGAAGACTTGTAGCACCTACAAGGTTAAATTCCCAATTTGCCCCCGT
ATAGTGTGCAAACCTATCGGCTGCGACTGCAAGGACGGCTTTGTATTCGATGACAATGTTAAAAAGTGCGTCTTGC
CACAGGATTGCACACCCATTTGCGAAGTtaatgaagaatattctccCTGCATCAATGGTGGATGCGGCCCTAAAAATTGC
AGCCAAGTTGGCCAGCCAAATATATGCGTGGATCCTGTAACTTGTATCAAGGGATGTGTCTGCGTCAAAGGGTAC
TTGAGAGCTTCAAATGGGACTTGCGTGCCAACTGACCAATGCCCACCAGCCATCTGCAAAGTTAATGAAGAATTAT
CTACCTGCATCAATGGCGGATGCGATCTTAGAAATTGCAGCCAGGTTGGCCAGCCAACTATCTGCGTAGATCCTGT
AACCTGTATCGTGGGATGTGTCTGCATCAAAGGGTACTTGAGAGCTTCGAATGGGACTTGCGTGCCAATTGACCA
ATGCCCAGTCCCTTCGAATGGCACGTGTGTCAACACGAGAAGTGATGTCAGCGCTGACTCCCAGTGCAATATGTCA
ACGCTGACCGATTGTAGTGTGAACAACAGTGACGTCACAACTACTACCTGTACAAACAGCCAGTACAACGGCGTT
GATATTACTGACTCGACAACTACTGGCACTACTCTCACAGGACCGGGATGCTCAGTTACCTCTTGCACTATTACTGG
AGGGATACCTGCCCCATCTGCTGCGTGTGTCATCTCTGGATGCATCGTGGGTGCCGCTTAG

Encoded protein

>NuZonadhesin-like
MWKTFVLLTCVVAIVSCNDYKCGPNEVFSNCTNGGCEPRNCSQAGKPVPCVKLDPKSCIKGCVCKPGYLRSANGTCVP
QNQCNEGCGGDPNAKPGCGYYCGKTCTTYETLLPICPRIPCKPNGCDCKDGYVYDGNVKKCVLPKDCIICGINEVYDVC
PAPCPPRRCDVDDRVIKCAAPPKPGDPQCKPGCRCADGYARNATGTCVLRKQCEQPLTCDRPNEIYDSCPAPCPGDSC
DVNPALVLCAPSPQYGDPTCKPACRCIDGYARNAAKKCVPRDQCHYKCGPNEVFSNCTNGDCEARNYSQAGKPVPCV
KLDPKSCIKGCVCQPGYLRSANGTCVPKNQCNEGCGNDPNSHPGCGVNCGRTCSTYKIKNVACILICYLNGCDCNDGF
VFDPNVKKCVLPKDCTPTCGLNEVYSSCINGGCGHRNCSQLGQPELCIDITPESCIKGCICASGYLRAANGTCIPQDKCPP
TGCGGDPNAQPGCGYNCGKTCSTYKVKFPICPRIVCKPIGCDCKDGFVFDDNVKKCVLPQDCTPICEVNEEYSPCINGG
CGPKNCSQVGQPNICVDPVTCIKGCVCVKGYLRASNGTCVPTDQCPPAICKVNEELSTCINGGCDLRNCSQVGQPTICV
DPVTCIVGCVCIKGYLRASNGTCVPIDQCPVPSNGTCVNTRSDVSADSQCNMSTLTDCSVNNSDVTTTTCTNSQYNGV
DITDSTTTGTTLTGPGCSVTSCTITGGIPAPSAACVISGCIVGAA

Note: the portion highlighted in grey corresponds to NuAFP-1 (without signal peptide); portions highlighted in green identify an imperfect hexapeptide repeat (A/SN/TGTCV/I), seven copies of which are found in this protein, including one at the N-terminus of NuAFP-1.

```
                   1        2    3    4         5           6   7 8         9     10
148-210            CGINEVYDVCPAP-CPPRRCDVDDRVIKCAAPPKPGDPQ-CKPGCRCADGYARNATGTCVLRKQC
22-82              CGPNEVFSNCTNGGCEPRNCSQAGKPVPCVKL----DPKSCIKGCVCKPGYLRSANGTCVPQNQC
283-343            CGPNEVFSNCTNGDCEARNYSQAGKPVPCVKL----DPKSCIKGCVCQPGYLRSANGTCVPKNQC
409-469            CGLNEVYSSCINGGCGHRNCSQLGQPELCIDI----TPESCIKGCICASGYLRAANGTCIPQDKC
600-658            CKVNEELSTCINGGCDLRNCSQVGQPTICV------DPVTCIVGCVCIKGYLRASNGTCVPIDQC
537-595            CEVNEEYSPCINGGCGPKNCSQVGQPNICV------DPVTCIKGCVCVKGYLRASNGTCVPTDQC
                   *  **  . *    *  .. .  ..   *       *   *  ** *  ** * :.***:  .:*
```

Alignment 1. CLUSTAL alignment of six "trypsin inhibitor-like" (TIL) cysteine-rich domains found in the *N. uddmanniana* "zonadhesin-like" protein whose coding sequence is located in the AFP locus of chromosome 6. TIL domains were identified using the NCBI Conserved Domain Search algorithm (https://www.ncbi.nlm.nih.gov/Structure/cdd/wrpsb.cgi). Numbers on the left refer to residue positions within the protein. TIL domains characteristically feature 10 cysteine residues (yellow shading) that form disulfide bonds in the pattern 1-7, 2-6, 3-5, 4-10 and 8-9. Cyan shading: hexapeptide imperfect repeat.

TIL domain

```
22-147             CGPNEVFSNCTNGGCEPRNCSQAGKPVPCVKL---DPKSCIKGCVCKPGYLRSANGTCVPQNQCN-EG--CGGDPNAKPG
148-282            CGINEVYDVCP-APCPPRRCDVDDRVIKCAAPPKPGDPQCKPGCRCADGYARNATGTCVLRKQCE-QPLTCDRPNEIYDS
283-408            CGPNEVFSNCTNGDCEARNYSQAGKPVPCVKL---DPKSCIKGCVCQPGYLRSANGTCVPKNQCN-EG--CGNDPNSHPG
409-536            CGLNEVYSSCINGGCGHRNCSQLGQPELCIDI---TPESCIKGCICASGYLRAANGTCIPQDKCPPTG--CGGDPNAQPG
                   ** ***:. *   . *  *. .  .:   *         .*  ** *  ** * *.***: :.:*      *.   :   .

22-147             CGYYC-GKTCTTYETLLPIC-----PRIPCKPNGCDCKDGYVYDGNVKKCVLPKDCI-I
148-282            CPAPCPGDSCDVNPALVLCAPSPQYGDPTCKP-ACRCIDGYARN-AAKKCVPRDQCHYK
283-408            CGVNC-GRTCSTYKIKNVAC-----I-LICYLNGCDCNDGFVFDPNVKKCVLPKDCTPT
409-536            CGYNC-GKTCSTYKVKFPIC-----PRIVCKPIGCDCKDGFVFDDNVKKCVLPQDCTPI
                   *   * * :* .       .         *    .* * **:. :  .****  .:*
```

Alignment 2. CLUSTAL alignment of four partial repeats containing TIL domains and distinct cysteine-rich regions found in the *N. uddmanniana* "zonadhesin-like" protein whose coding sequence is located in the AFP locus of chromosome 6. Numbers on the left refer to residue positions within the protein. Cysteine residues (yellow shading) and the hexapeptide imperfect repeat (cyan shading) are highlighted.

```
1                  VPSNGTCVNTRSDVSADSQCNMSTLTDCSVNNSDVTTTTCTNSQYNGVDITDSTTTGTTL
6                  RASNGTCV-------PTDQC-PPAICKVNEELSTCINGGCDLRNCSQVGQPTICVDP---
3                  RNATGTCV-------LRKQCEQPLTCDRPNEIYDSCPAPCPGDSCDVNPALVLCAPSPQY
4                  RSANGTCV-------PKNQC--NEGCGNDPNSHPGCGVNC-GRTCSTYKIKNVACI----
5                  RAANGTCI-------PQDKC-PPTGCGGDPNAQPGCGYNC-GKTCSTYKVKFPICPR---
2                  RSANGTCV-------PQNQC--NEGCGGDPNAKPGCGYYC-GKTCTTYETLLPICPR---
                     :.***:       .:*          :         *

1                  TGPGCSVTSCTITGGIPAPSAA--CVI-SGCIVGAA-----------
6                  --VTCIV-GCVCIKGYLRASNGT-CVPIDQCP---------------
3                  GDPTCKPA-CRCIDGYARN-AAKKCVPRDQCHYKCGPNE--------
4                  --LICYLNGCDCNDGFVFDPNVKKCVLPKDCTPTCGLNEVYSSCING
5                  --IVCKPIGCDCKDGFVFDDNVKKCVLPQDCTPICEVNEEYSPCIN-
2                  --IPCKPNGCDCKDGYVYDGNVKKCVLPKDCI-ICGINEVYDVCPAP
                       *    *    *         **  . *
```

Alignment 3. CLUSTAL alignment of five cysteine-rich partial repeats within the *N. uddmanniana* "zonadhesin-like" protein, plus NuAFP-1. The hexapeptide imperfect repeat (cyan shading, red box) was used to anchor the sequences selected for alignment, where sequence #1 is NuAFP-1. Residues highlighted in cyan are identical to the corresponding residues in NuAFP-1, while those highlighted in green are similar and those highlighted in grey show either limited or no similarity. Aligned cysteines are highlighted in yellow.

```
NuAFP-1      SNGTCVNTRSDVSADSQCNMSTLTDCSVNNSD
CfAFP-9      SDGTCTNTNSQFA-NSQCVTSTLTDCNIRDSQ
CfAFP-8      SDGTCVNMNSQIS-NSRCVDTTATNCYIDNSQ
CfAFP-11     SDGTCVSTNSQIS-NSRCVNSTANNCYIDNSQ
NuAFP-2      SDGTCTNTNSQISADSQCTTSTLTDCSLDNSN
NuAFP-4      SDGTCVNTRSFLSANSRCTSSTLTDSHVDNSN
NuAFP-7      SDGLCVNTQSHLSADSKCVTSTLTSCSVDNSD
NuAFP-3      SDVPCVNTNSQLSANSQCTSSTLTNCHVNNSK
CfAFP-1      PDGTCVSTNSYISTNSQCFSSTVTNSYINNSQ
CfAFP-6      SDGSCVSTNSWISPNSQCVRSTLTNCNLDNSQ
CfAFP-5      SDGTCTYNNSHIT-NTQCVTSTLNNCNVRDSQ
CfAFP-15     SDGSCTKTNTQFT-NSQCVRSTLTNCNVRNSQ
CfAFP-16     SDGSCTNTNSQIT-NSQCVTSTLTNCNLRNSQ
CfAFP-3      SDGTCRNTNSQISANSLCATSTLTNCYIDDSQ
CfAFP-2      SDGSCVNTNSQVSENSWCVRSTLTFCNIDNSQ
CfAFP-4      SDGTCRNTNSQIT-NSQCVDSVPTNCYIDNSE
CfAFP-12     SDGTCTNTNSQLSANSKCDKSTLTNCYVDKSE
CfAFP-7      SDGTCVNTNSQITANSQCVKSTATNCYIDNSQ
CfAFP-13     SDGTCTKTNSWISPNSQCVRSTLTNCNVDNSQ
CfAFP-14     SDGTCTSTNSQISRNSRCVKSTVTNCYIDNSQ
```

Alignment 4. CLUSTAL alignment of an N-terminal portion of CfAFPs and NuAFPs, showing the presence of the imperfect hexapeptide repeat found in NuAFP-1 (SNGTCV) in all other AFPs. Within the hexapeptide repeat, residues highlighted in blue are identical to the aligned residues in NuAFP-1, while those highlighted in green are similar and those highlighted in grey show little or no similarity.

```
Query  22    CGPNEVFSNCTNGGCEPRNCSQAGKPVPC--VKLDPKSCIKGCVCKPGYLRSANGTCVPQ  79
             C PNE +S+C N GC  RNCSQ G P+ C  V +  + CI+G +CKPGY R+ NGTC+P
Sbjct  338   CKPNEEYSDCINRGCYARNCSQLGYPLACADVVVKDEDCIEGFICKPGYTRADNGTCIPT  397

Query  80    NQCNEGCGGDPNAKPGCGYYCGKTCTTYETLLPICPRIPCKPNGCDCKDGYVYDGNVKKC  139
             +QC   CGGDPNA+ GCG +C K C+ Y+    ICP I C  NGC C++GY+YD N KKC
Sbjct  398   DQC-PSCGGDPNAESGCGSFC-KRCSNYKNESLICP-IGCILNGCQCREGYIYDQNTKKC  454

Query  140   VLPKDCI-ICGINEVYDVCP-APCPPRRCDVDDRVIKCAAPPKPGDPQ-CKPGCRCADGY  196
             VLPK C   C INEVY  C    C  + C      + C       DP+ CK GC C  GY
Sbjct  455   VLPKQCTPTCKINEVYSECTNTGCTAQNCSQLGTPVPCVRL----DPKYCKKGCVCDAGY  510

Query  197   ARNATGTCVLRKQCEQPLTCDRPNEI------------YDSCPAPCPGDSCDVNPALVLC  244
              R   GTC+  K+C  P     PN              Y S P  CP D C +N
Sbjct  511   LRADNGTCIPEKEC--PACGGDPNAESGCGTFCETCTNYKSGPVACP-DICLLN------  561

Query  245   APSPQYGDPTCKPACRCIDGYARNA-AKKCVPRDQCHYKCGPNEVFSNCTNGDCEARNYS  303
                           C+C  GY  +   KKCV  +QC   C  NEV+S+C N  C+ RN S
Sbjct  562   -------------GCQCRKGYIYDPNIKKCVLPEQCTPTCKANEVYSDCNNSGCDPRNCS  608

Query  304   QAGKPVPCVKLDPKSCIKGCVCQPGYLRSANGTCVPKNQCNEGCGNDPNSHPGCGVNCGR  363
             Q G PVPCV + P SCIKGCVC  G+LR+ NGTC+P+ QC   CG DPN+  GCG  C
Sbjct  609   QLGNPVPCVDISPDSCIKGCVCASGFLRADNGTCIPEKQC-PACGGDPNAESGCGTFC-E  666

Query  364   TCSTYKIKNVACILICYLNGCDCNDGFVFDPNVKKCVLPKDCTPTCGLNEVYSSCINGGC  423
             TC+ YK   VAC  IC LNGC C +G+++DPN+KKCVLP+ CTPTC  NEVYS CIN GC
Sbjct  667   TCTNYKSGPVACPDICVLNGCQCKEGYIYDPNIKKCVLPEQCTPTCKANEVYSDCINSGC  726

Query  424   GHRNCSQLGQPELCIDITPESCIKGCICASGYLRAANGTCIPQDKCPPTGCGGDPNAQPG  483
               RNCSQLG P  C+DI+P++CIKGC+CASGYLRA NGTCIP+ +CP   CGGDPNA+ G
Sbjct  727   DPRNCSQLGNPVACVDISPDACIKGCVCASGYLRADNGTCIPEKQCP--SCGGDPNAESG  784

Query  484   CGYNCGKTCSTYKVKFPI-CPRIVCKPIGCDCKDGFVFDDNVKKCVLPQDCTPICEVNEE  542
             CG  C       +   PI CPRI C P  C CK G+++D N+KKCVLP  CTP C+ NEE
Sbjct  785   CGVFCD------RCSGPIPCPRI-CYPNSCQCKSGYIYDQNIKKCVLPNQCTPTCKDNEE  837

Query  543   YSPCINGGCGPKNCSQVGQPNICVD--PVTCIKGCVCVKGYLRASNGTCVPTDQCP----  596
             YS CINGGCGP+NCSQ+G P  CVD  P  CIKGCVC  G+LRA NGTC+P  QCP
Sbjct  838   YSDCINGGCGPRNCSQLGNPVACVDISPDACIKGCVCASGFLRADNGTCIPEKQCPSCGD  897

Query  597   ---------------------PAIC------------------------------KVNEE  605
                                  P IC                              K NEE
Sbjct  898   DPNAESGCGVFCDRCSGPIKCPRICYPNGCQCKSGYIYDQNIKKCVLPNQCTPTCKDNEE  957

Query  606   LSTCINGGCDLRNCSQVGQPTICV--DPVTCIVGCVCIKGYLRASNGTCVPIDQCP  659
              S CINGGCD RNCSQ+G P  CV  +P  CI GCVC  GYLRA NGTC+P  QCP
Sbjct  958   YSDCINGGCDARNCSQLGNPIPCVKLNPDDCIKGCVCSSGYLRADNGTCIPEKQCP  1013
```

Alignment 5. Sequence alignment output of a blastp search against the NCBI NR database using the "zonadhesin-like" protein identified in *N. uddmanniana* Chr. 6 as query: closest hit is "zonadhesin-like isoform X5" [*Ostrinia furnacalis*] XP_028162860.1. 2e -169. Presence of the imperfect hexapeptide repeat in both proteins is highlighted in blue. Please note that the portion of the protein corresponding to NuAFP-1 was not included in the blastp output.

```
>g2410.t2
          Length = 1494

 Score =  754 bits (1948), Expect = 0.0
 Identities = 376/742 (50%), Positives = 447/742 (60%), Gaps = 41/742 (5%)

Query: 16  SCNDYKCGPNEVFSNCTNGGCEPRNCSQAGKPVPCVKLDPKSCIKGCVCKPGYLRSANGT 75
            S   +KCG NEVFSNCTNGGC+ R CSQAGKPVPCVKLDPKSC KGCVC+PGYLR++NGT
Sbjct: 102 SALSFKCGANEVFSNCTNGGCDARYCSQAGKPVPCVKLDPKSCKKGCVCQPGYLRASNGT 161

Query: 76  CVPQNQCNEGCGGDPNAKPGCGYYCGKTCTTYETLLPICPRIPCKPNGCDCKDGYVYDGN 135
            CVP+NQCNEGCG DPNA PGCG  CGKTC++Y+   PICP + C  NGCDC +GYV+D
Sbjct: 162 CVPRNQCNEGCGDDPNAHPGCGVNCGKTCSSYKAKNPICP-LFCNRNGCDCNEGYVFDPA 220

Query: 136 VKKCVLPKDC-IICGINEVYDVCP-APCPPRRCDVDDRVIKCAAPPKPGDPQCKPGCRCA 193
            V KCV+P+DC ++CG+NEV   C    C P  C      +KC  P K     C  GC C
Sbjct: 221 VNKCVIPEDCTLVCGVNEVASNCANGGCGPLTCAGVGSPVKCVKPIK-----CNKGCVCL 275

Query: 194 DGYARNATGTCVLRKQCEQPLTCDRPNEIYDSCPAPCPGDSCDVNPALVLCAPSPQYGDP 253
            D Y R+  GTC+ +  C   +TC+        C   C G +C          P
Sbjct: 276 DYYVRSDNGTCIPQGDC-PAVTCNGDPNAKPGCGVNC-GKTCSTYKIKNPVCPD------ 327

Query: 254 TCKP-ACRCIDGYARNA-AKKCVPRDQCHYKCGPNEVFSNCTNGDCEARNYSQAGKPVPC 311
             CK   C C DG+  +A   KCV    C   CGPNE F +C    C   N SQ G+P  C
Sbjct: 328 ICKAFGCDCKDGFVFDANLNKCVLPKDCTPICGPNERFDSCAGFGCGRWNCSQLGQPELC 387

Query: 312 VKLDPKSCIKGCVCQPGYLRSANGTCVPKNQCNEGCGNDPNSHPGCGVNCGRTCSTYKIK 371
            +   P  C  GC C  GYLR+ NGTCVP++QC  GC  DPN+ PGCGVNCGRTCSTYK+K
Sbjct: 388 IDPAPGGCKNGCTCVNGYLRADNGTCVPQDQC-PGCNGDPNAEPGCGVNCGRTCSTYKLK 446

Query: 372 NVACILICYLNGCDCNDGFVFDPNVKKCVLPKDCTPTCGLNEVYSSCINGGCGHRNCSQL 431
            NVAC  IC LNGCDC  G+VFD N++KCV+P++C+P C LNE + SC+  GCG RNCSQL
Sbjct: 447 NVACPAICALNGCDCKKGYVFDNNLQKCVMPQECSPICRLNERFDSCVGFGCGRRNCSQL 506

Query: 432 GQPELCIDITPESCIKGCICASGYLRAANGTCIPQDKCPPTGCGGDPNAQPGCGYNCGKT 491
            GQPELCID  P SC  GC C  GYLRA NGTC+PQD+CP TGC GDPNA+PGCG NCGKT
Sbjct: 507 GQPELCIDPAPGSCKNGCTCVDGYLRAENGTCVPQDQCPATGCNGDPNAKPGCGANCGKT 566

Query: 492 CSTYKVKFPICPRIVCKPIGCDCKDGFVFDDNVKKCVLPQDCTPICEVNEEYSPCINGGC 551
            CSTYK+K P CP I C   GCDC DGFVFD N+KKCVLP+DCTPIC +NE +  C   GC
Sbjct: 567 CSTYKIKNPGCPAI-CSVNGCDCNDGFVFDANLKKCVLPKDCTPICGLNERFDSCAGFGC 625

Query: 552 GPKNCSQVGQPNICVDPVT--CIKGCVCVKGYLRASNGTCVPTDQCPPAICKVNEELSTC 609
            G  NCSQ+GQP +C DP+   C  GC C+KGYLRA NGTCVP DQCP      N +
Sbjct: 626 GRWNCSQLGQPELCRDPIPGGCKNGCTCIKGYLRAGNGTCVPEDQCPGCNGDRNAKPGCG 685

Query: 610 INGGCDLRNCSQVGQPTICVDPVTCIVGCVCIKGYLRASN-GTCVPIDQCPVPSNGTCVN 668
            +N G   R CS      +    +  I GC C  GY+   N   CV  +QC     G
Sbjct: 686 LNCG---RTCSTYKLKNVACSTICQINGCDCKDGYVFDDNVRKCVLPEQCSEGCGG---- 738

Query: 669 TRSDVSADSQCNMSTLTDCSV--NNSDVTTTTCTNSQYNGVDITDSTTTGTTLTGPGCSV 726
               D +A   C ++    CS     + +    C   +  G D  D       L    C +
Sbjct: 739 ---DPNAHPGCGVNCGKTCSTYKTKNPICPRIC---KVGGCDCNDGYVYDANLN--KCVM 790

Query: 727 TS-CTITGGIPAPSAACVISGC 747
               CT   G+     +C  +GC
Sbjct: 791 PEYCTPICGLNEQYDSCTNAGC 812
```

Alignment 6. Sequence alignment output of a local blastp search against the proteome of *Apotomis turbidana* Chr. 4 (BRAKER2 annotation) using the “zonadhesin-like” protein identified in *N. uddmanniana* Chr. 6 as query. The *A. turbidana* protein (g2410.t2, a zonadhesin-like) is larger than the *N. uddmanniana* protein, and shows limited similarity to the latter in the region that corresponds to NuAFP-1 (yellow shading). Please note that the gene encoding the *A. turbidana* protein is found in a locus believed to be homologous to the NuAFP locus on *N. uddmanniana* chromosome 6.

Supplementary file 8 – *Choristoneura fumiferana* transcriptome assembly

Different *C. fumiferana* life stages and tissues were used for the purpose of generating a transcriptome assembly. With one exception, all insects came from the standard diapause strain of the spruce budworm (Roe et al. 2018) reared at the Insect Production and Quarantine Laboratories (IPQL) (https://www.nrcan.gc.ca/science-and-data/research-centres-and-labs/forestry-research-centres/great-lakes-forestry-centre/insect-production-and-quarantine-laboratories/13467) of the Great Lakes Forestry Centre, Sault Ste. Marie, ON, Canada. The other group of larvae came from a diapause-free strain (Harvey 1957), also reared at the IPQL.

Total RNA was extracted using the Trizol reagent, following the manufacturer's RNA isolation protocol. Each individual sample was checked for quality using the Experion Analysis Kit (Bio-Rad). For eggs and early instars, RNA was extracted from a pool of 10-60 insects (eggs: 0.05-0.1 g/1 mL Trizol; L1 and L2 larvae: 0.01-0.03 g/1 mL Trizol) reared at 22°C (16h light:8h dark, 55% RH). For later life stages, extractions were performed on small pools of insects or tissues (see list below). Illumina RNA-seq libraries were prepared at the Plateforme d'Analyses Génomiques of the Institut de Biologie Intégrative et des Systèmes (Université Laval, Canada) and submitted for sequencing at the McGill University and Genome Quebec Innovation Centre (Montreal, Canada). Prior to assembly, raw read adapters were removed using Trimmomatic V0.39. Then, the transcriptome was assembled using Trinity v2.13.2 (https://github.com/trinityrnaseq/trinityrnaseq/releases). Residual adapters remaining in the Trinity output were removed using a custom-made program that targets adapters according to their hit position in a blast search on Univec. The transcriptome and RNA-seq reads used to assemble it have been deposited at the NCBI under BioProject PRJNA750049.

List of insects used for RNA extraction:

1. Eggs (days 1, 3 and 5), 1st instar (days 1, 3 and 5), 2nd instar (days 1, 3 and 5) of the standard diapause strain.
2. Eggs (days 1, 3 and 5), 1st instar (days 1, 3 and 5), 2nd instar (days 1, 3 and 5) of the diapause-free strain.
3. Twenty L6D3 larval heads; 2 male + 2 female L5s molting to L6; 2 male and 2 female L6D2.
4. Adult female antennae, adult female pheromone gland, adult female brain, adult female reproductive tract, adult male antennae, adult male brain, adult male reproductive tract.

Harvey GT (1957) The occurrence and nature of diapause-free development in the spruce budworm, *Choristoneura fumiferana* (Clem.) (Lepidoptera: Tortricidae). *Can J Zool* **35,** 549-572.

Roe **AD**, Demidovich **M**, Dedes **J** (2018) Origins and history of laboratory insect stocks in a multispecies insect production facility, with the proposal of standardized nomenclature and designation of formal standard names. *J Insect Sci* **18(3),** 1.

BUSCO results for transcriptome (dataset lepidoptera_odb10)

C:97.6%[S:24.8%,D:72.8%],F:1.5%,M:0.9%,n:5286

5160	Complete BUSCOs (C)
1310	Complete and single-copy BUSCOs (S)
3850	Complete and duplicated BUSCOs (D)
80	Fragmented BUSCOs (F)
46	Missing BUSCOs (M)
5286	Total BUSCO groups searched

Supplementary file 9 – phylogenetic analyses of antifreeze proteins

We conducted a series of phylogenetic analyses using nucleotide and amino acid sequences under maximum likelihood (ML) and Bayesian inference (BI) approaches implemented in IQ-TREE v. 1.6.12 available on the web server (Nguyen et al., 2015; Trifinopoulos et al., 2016) and MrBayes v.3.2.7 (Ronquist et al., 2012). For the first set of analyses, we used data matrices with concatenated sequence data for 14 AFPs found across the *Choristoneura* group with species set as the terminal taxa; in the second set of analyses, we used AFPs as terminal taxa in data matrix including sequences for 14 AFPs for each *Choristoneura* species; finally, we conducted additional set of analyses with AFPs as terminal taxa and including sequence data for an outgroup taxon, *Notocelia uddmanniana*; this set of analyses included only 4 AFPs (AFP-3, 4, 7, 12), for which we could reliably determine homology between the AFPs found in the outgroup and in the *Choristoneura* group.

For the maximum likelihood analysis, best fit model was determined using ModelFinder (Kalyaanamoorthy et al., 2017) available in IQ-TREE. The best partitioning scheme and substitution model for the Bayesian inference analysis was determined using PartitionFinder2 (Lanfear et al., 2017).

Maximum likelihood analysis of the nucleotide sequences was performed using the TIM substitution model with gamma-distributed across-site rate heterogeneity (+G4) and empirical base frequencies (+F). Branch support was estimated using the ultrafast bootstrap and SH-aLRT test with 1000 replicates each. Amino acid sequences were analyzed using the JTTDCMut model with gamma-distributed across-site rate heterogeneity selected under the Bayesian information criterion by ModelFinder.

Bayesian inference analyses were conducted using the GTR model and partitioning by codon position for the nucleotide sequence data and using the WAG model for the amino acid sequence data. For both nucleotide and amino acid data, we modeled across-site rate heterogeneity using gamma distribution with four discrete categories. For each analysis, we performed four independent Metropolis-coupled Markov chain Monte Carlo (MCMCMC) runs with four chains of 10 million iterations each. Parameter values were sampled every 1,000 generations. Independent runs were checked for convergence and stationarity in Tracer 1.7 (Rambaut et al., 2014) as well as using the standard output from MrBayes runs (average standard deviation of split frequencies < 0.01, potential scale reduction factor $\cong 1$, and effective sample size for each parameter > 200). The initial 25% of each MCMCMC run were discarded as burn-in. Final results were summarized as majority rule consensus trees with branch support values representing posterior probabilities.

References:

Nguyen L-T, Schmidt HA, von Haeseler A, Minh BQ (2015) IQ-TREE: A Fast and Effective Stochastic Algorithm for Estimating Maximum-Likelihood Phylogenies. *Molecular biology and evolution* **32,** 268–274. pmid:25371430

Trifinopoulos J, Nguyen L-T, von Haeseler A, Minh BQ (2016) W-IQ-TREE: a fast online phylogenetic tool for maximum likelihood analysis. Nucleic Acids Research **44,** 232–235.

Ronquist F, Teslenko M, van der Mark P, Ayres DL, Darling A, Höhna S, et al. (2012) MrBayes 3.2: efficient Bayesian phylogenetic inference and model choice across a large model space. Syst Biol. **61,** 539–42. pmid:22357727

Kalyaanamoorthy S, Minh BQ, Wong TK, Von Haeseler A, Jermiin LS (2017) ModelFinder: fast model selection for accurate phylogenetic estimates. *Nature methods* **14,** 587-589.

Lanfear R, Frandsen PB, Wright AM, Senfeld T, Calcott B (2017) PartitionFinder 2: new methods for selecting partitioned models of evolution for molecular and morphological phylogenetic analyses. *Molecular biology and evolution*, **34,**772-773.

Rambaut A, Suchard MA, Xie D, Drummond AJ (2014) Tracer v1.7, Available from http://beast.bio.ed.ac.uk/Tracer.

Supplementary trees

In reference to fig. 2: comparison of tree topologies using either nucleotide or amino acid sequences and either maximum likelihood or Bayesian inference to construct the trees.

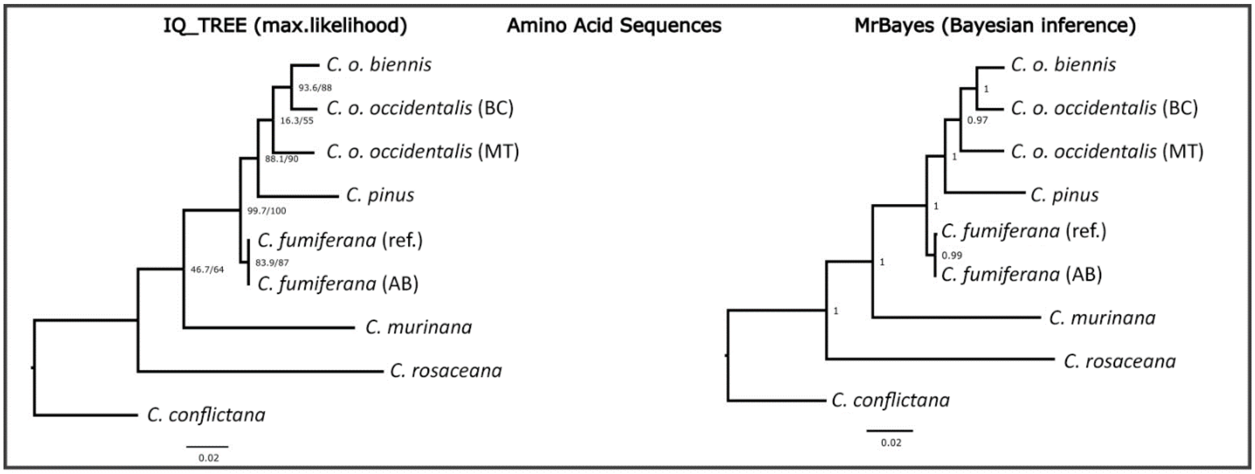

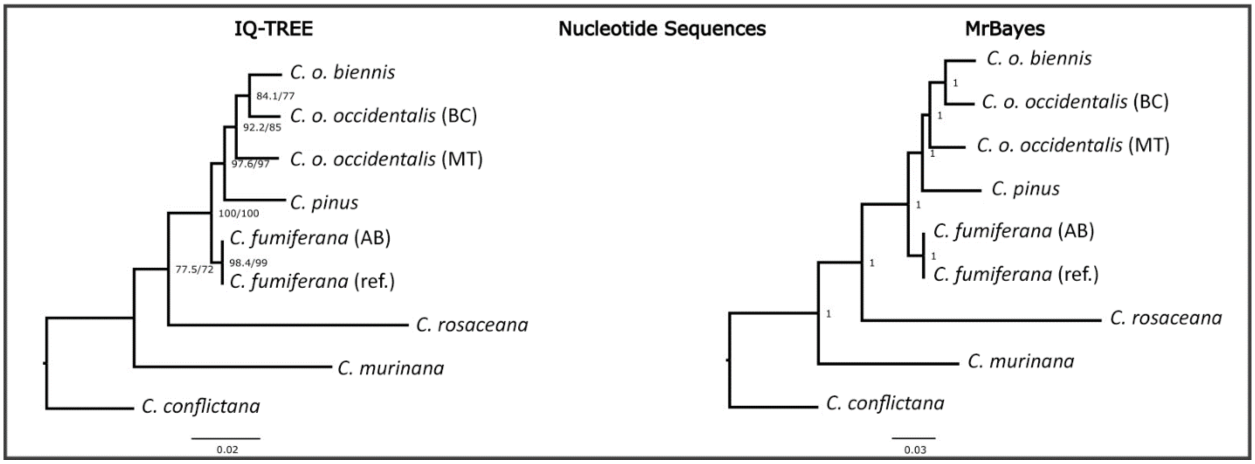

In reference to fig. 3: comparison of tree topologies using either nucleotide or amino acid sequences and either maximum likelihood or Bayesian inference to construct the trees.

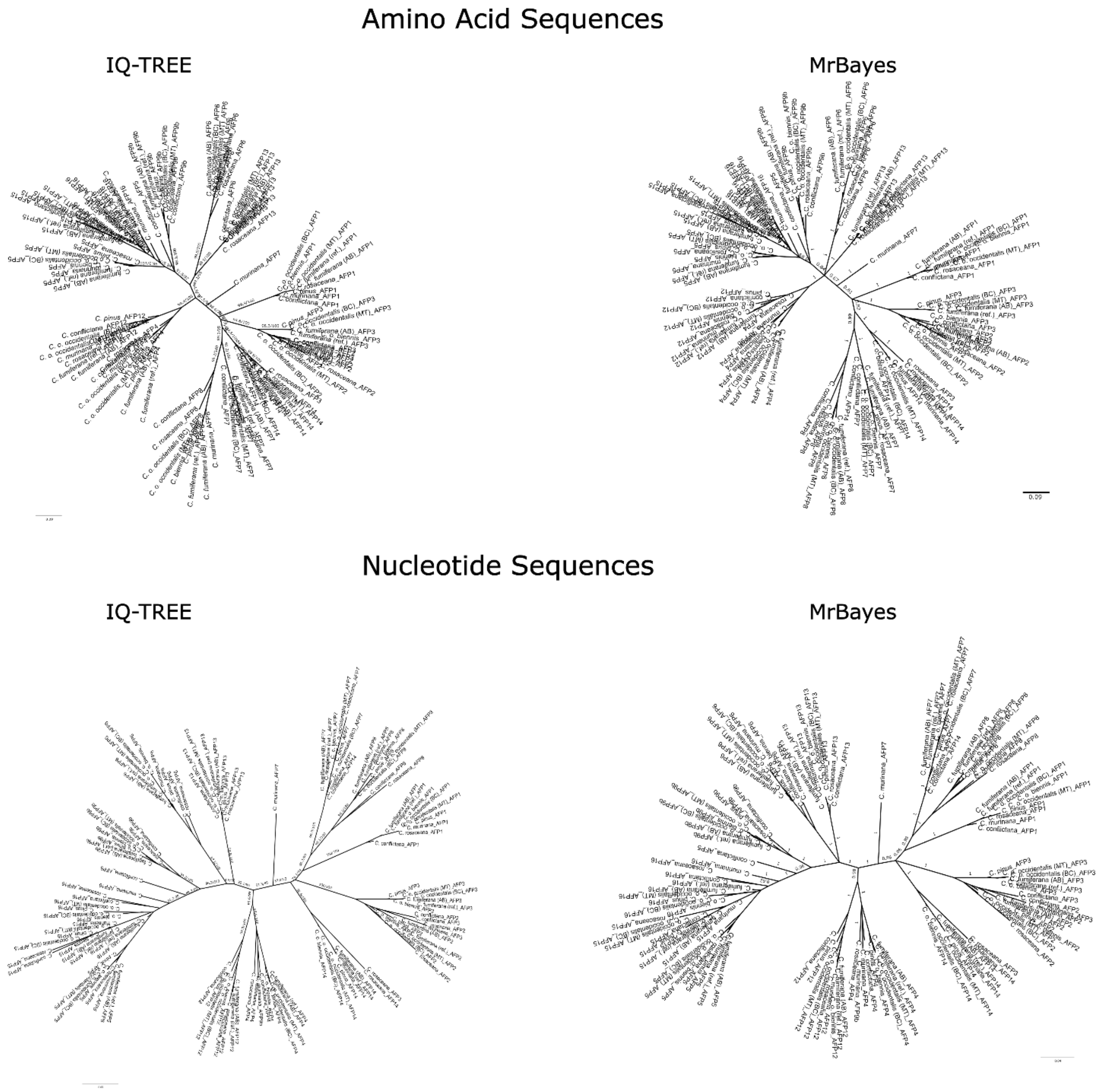

In reference to Supplementary fig. 4: comparison of tree topologies using either nucleotide or amino acid sequences and either maximum likelihood or Bayesian inference to construct the trees.

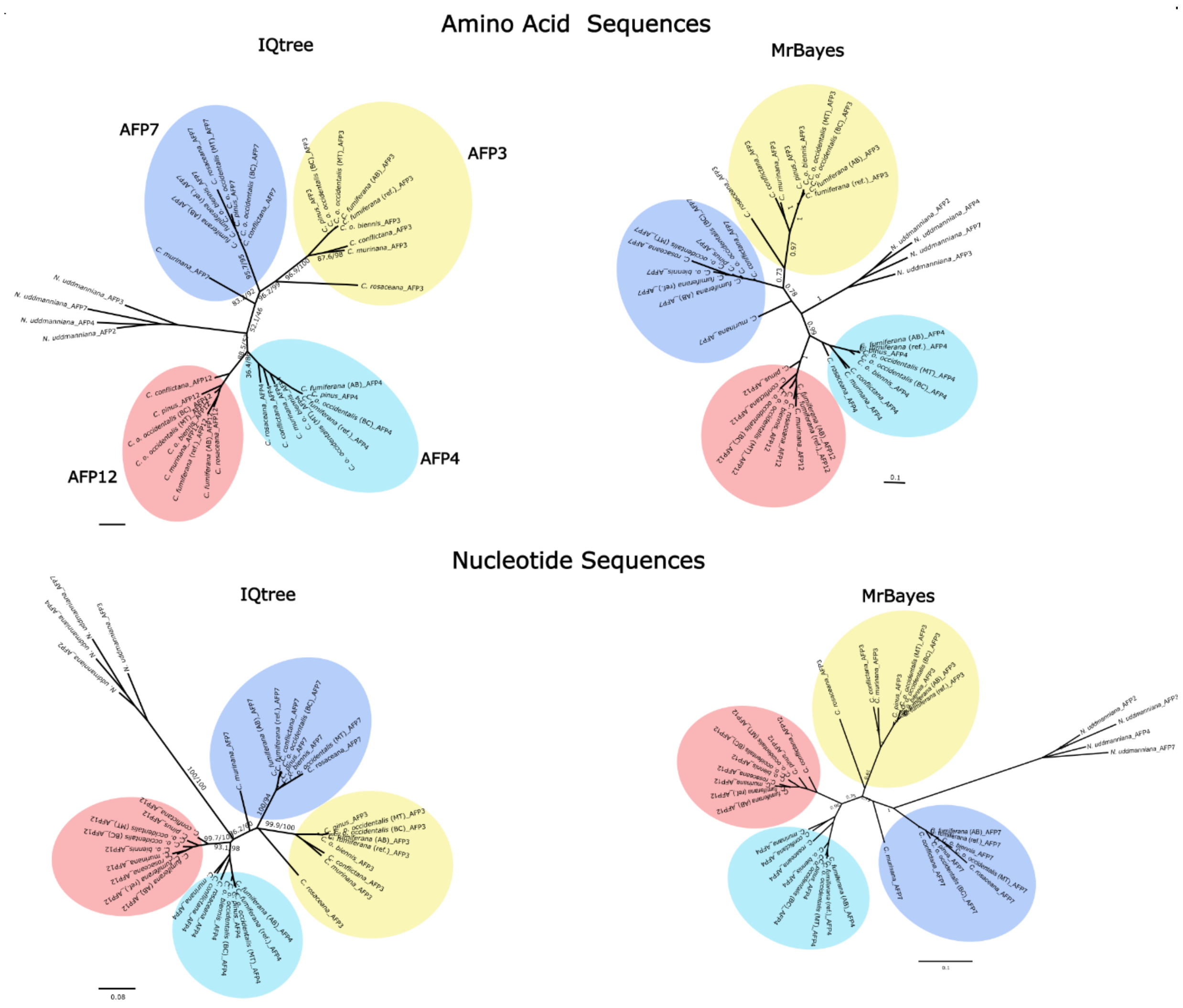

Supplement: evac087_Supplementary_Data [file evac087_supplementary_data.zip › Supplementary files 4, 6, 8 and 9 - revised.pdf]
