# Supplementary material for: The Spruce Budworm Genome: Reconstructing the Evolutionary History of Antifreeze Proteins
Source: Genome Biol Evol. 2022 Jun 7;14(6):evac087. doi: 10.1093/gbe/evac087 (PMC9210311; doi:10.1093/gbe/evac087)
Supplement: evac087_Supplementary_Data [file evac087_supplementary_data.zip › Supplementary tables.pdf]

Supplementary tables for the manuscript entitled:

The Spruce Budworm Genome: Reconstructing the Evolutionary History of Antifreeze Proteins

by

Béliveau C, Gagné P, Picq S, Vernygora O, Keeling CI, Pinkney K, Doucet D, Wen F, Johnston JS, Maaroufi H, Boyle B, Laroche J, Dewar K, Juretic N, Blackburn G, Nisole A, Brunet B, Brandao M, Lumley L, Duan J, Quan G, Lucarotti CJ, Roe AD, Sperling FAH, Levesque RC, Cusson M.

For: *Genome Biology and Evolution*

Supplementary table 1: Repetitive content of *Choristoneura fumiferana* genome assembly.. p. 2

Supplementary table 2: qPCR primers used to quantify accumulation of CfAFP-11 and CfAFP-13 transcripts…………………………….........………………………….. p. 3

Supplementary table 1. Repetitive content of *Choristoneura fumiferana* genome assembly[1], as determined using RepeatModeler/RepeatMasker.

	No. of elements[2]	Length occupied	% of sequence
Retroelements	397,477	78,115,850 bp	13.71 %
SINEs	0	0 bp	0.00 %
Penelope	9,890	1,967,831 bp	0.35 %
LINEs	190,067	32,170,918 bp	5.65 %
CRE/SLACS	20,244	2,936,601	0.52 %
L2/CR1/Rex	35,832	7800345 bp	1.37 %
R1/LOA/Jockey	2,756	1,693,204 bp	0.30 %
R2/R4/NeSL	1,147	301,721 bp	0.05 %
RTE/Bov-B	105,075	14,804,293 bp	2.60 %
L1/CIN4	0	0 bp	0.00 %
LTR elements	207,410	45,944,932 bp	8.07 %
BEL/Pao	9,666	6,494,342 bp	1.14 %
Ty1/Copia	8,548	3,743,760 bp	0.66 %
Gypsy/DIRS1	18,624	8,946,676 bp	1.57 %
Retroviral	0	0 bp	0.00 %
DNA transposons	15,127	3,674,146 bp	0.65 %
hobo-Activator	1,852	217,985 bp	0.04 %
Tc1-IS630-Pogo	7,140	1,861,720 bp	0.33 %
En-Spm	0	0 bp	0.00 %
MuDR-IS905	0	0 bp	0.00 %
PiggyBac	77	30,479 bp	0.01 %
Tourist/Harbinger	935	114,793 bp	0.02 %
Other (Mirage, P-element, transib)	96	35,080 bp	0.01 %
Rolling-circles	0	0 bp	0.00 %
Unclassified	1410730	208,101,637 bp	36.54 %
**Total interspersed repeats**		289,891,633 bp	**50.90 %**
Small RNA	0	0 bp	0.00 %
Satellites	0	0 bp	0.00 %
Simple repeats	96,426	4,397,263 bp	0.77 %
Low complexity	10,525	490,465 bp	0.09 %
**Total bases masked**		294,779,361 bp	**51.75 %**

[1] G+C content: 37.83 %.

[2] Most repeats fragmented by insertions or deletions have been counted as one element

Supplementary table 2. qPCR primers used to quantify accumulation of CfAFP-11 and CfAFP-13 transcripts in spruce budworm eggs, 1st instars and 2nd instars of both diapause and diapause-free strains

Primer name	Primer sequence
CfAFP-11 F1	GCC AGG TCG AGA CCA CTA C
CfAFP-11 R1	GAC GCC GTT GAA TTG GCT G
CfAFP-13 F1	CGA TAC AAC GGC ATC TAT ATA ACG TCT
CfAFP-13 R1	TCA GTG TAC ATC CAG AGA TCT TGC
